# Microscopic Insights into Magneto-optics and Magneto-transport in 2D Perovskites

**DOI:** 10.1021/acs.nanolett.6c00182

**Published:** 2026-04-16

**Authors:** Roberto Rosati, Jonas K. König, Sophia Terres, Joshua J. P. Thompson, Michał Baranowski, Paulina Płochocka, Alexey Chernikov, Ermin Malic

**Affiliations:** ‡ Department of Physics, 9377 Philipps-Universität Marburg, Renthof 7, D-35032 Marburg, Germany; § mar.quest/Marburg Center for Quantum Materials and Sustainable Technologies, Hans-Meerwein-Straße 6, D-35032 Marburg, Germany; ¶ Institute of Applied Physics and Würzburg−Dresden Cluster of Excellence ct.qmat, 9169 TUD Dresden University of Technology, 01069 Dresden, Germany; ⊥ Department of Materials Science and Metallurgy, 2152 University of Cambridge, CB2 1TN Cambridge, U.K.; ∥ Department of Experimental Physics, Wrocław University of Science and Technology, Wybrzeże Wyspiańskiego 27, 50-370 Wrocław, Poland; ∇ Laboratoire National des Champs Magnétiques Intenses, EMFL, CNRS UPR 3228, Université Grenoble Alpes, 38042 Grenoble, France; ○ Laboratoire National des Champs Magnétiques Intenses, EMFL, CNRS UPR 3228, Université Toulouse, Université de Toulouse 3, INSA-T, 31400 Toulouse, France

**Keywords:** Two-dimensional perovskites, Excitons, Magnetic field, Magneto-optics, Magneto-transport, Exciton fine structure

## Abstract

Two-dimensional (2D) perovskites host tightly bound excitons with a fine structure shaped by spin–orbit coupling and exchange interaction. In the prototypical 2D perovskite, (PEA)_2_PbI_4_, the dark exciton lies below two bright excitons and a gray state. In-plane magnetic fields split the bright excitons, polarize them longitudinally (L) and transversely (T) to the field, and hybridize them with gray and dark states, respectively. The impact of magnetic fields on exciton emission and diffusion has remained largely unexplored. Here, we investigate time- and temperature-resolved magneto-optics and magneto-transport of excitons in (PEA)_2_PbI_4_. We demonstrate that magnetic fields enhance emission from X_T_ excitons and accelerate the transport of X_L_ states. We trace this back to suppressed scattering of X_T_ states into dark excitons, while X_L_ states form highly mobile hot dark excitons. The acquired microscopic insights offer guidance for experimental studies on magneto-optics and magneto-transport in layered perovskites.

## Graphical abstract

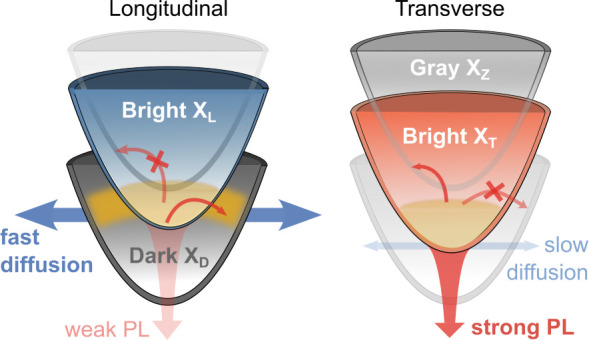

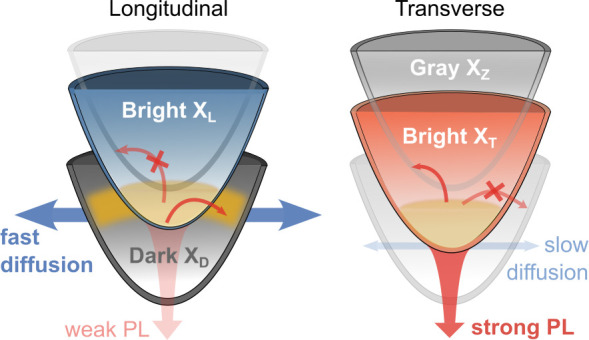

The investigation of organic–inorganic metal halide perovskites over the past decade, driven by their remarkable potential for energy harvesting and light emission,^1–5^ has triggered a broad exploration of their structural derivatives.^6–10^ Among numerous classes of materials, two-dimensional (2D) Ruddlesden–Popper perovskites have attracted much attention because they are characterized by considerably improved environmental stability,^11^ reasonable power conversion efficiency,^12–14^ and superior light emission properties.^7,15^ Inherent quantum and dielectric confinement strongly enhances excitonic effects,^16–19^ similar to the case of transition-metal dichalcogenides (TMDs).^20–23^ The interplay between strong spin–orbit coupling, crystal-field splitting, and electron–hole exchange interaction in 2D perovskites leads to a distinctive exciton fine structure.^24–28^ In the prototypical orthorhombic 2D perovskite, (PEA)_2_PbI_4_, there is a low-lying dark exciton state (X_D_), two nearly degenerate in-plane-polarized bright states (X_B_±__), and an out-of-plane-polarized gray state (X_Z_) (Figure 1a).^25–29^ The interplay between this fine structure and exciton–phonon scattering is responsible for the emergence of a phonon bottleneck, which facilitates highly efficient light emission,^30^ although the ground state in these materials is dark and bright–dark exciton splitting can reach tens of millielectronvolts.^26,29,31^

**Figure 1:**
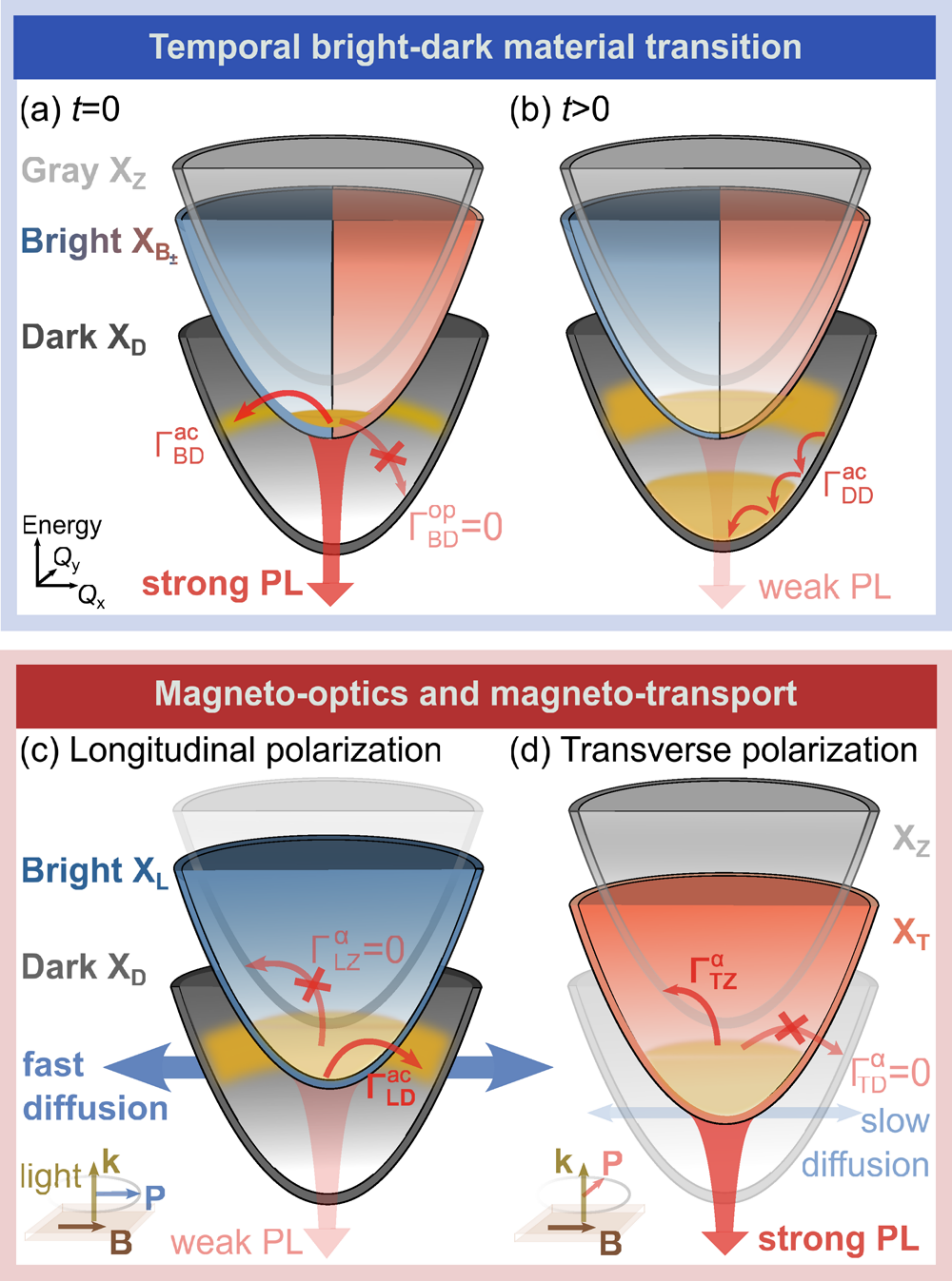
Exciton emission and diffusion. Sketch of the exciton fine structure in the monolayer (PEA)_2_PbI_4_ perovskite including bright, dark, and gray states as well as their occupation (a) shortly after optical excitation (*t* = 0) and (b) after a delay time (*t* > 0, yellow shaded). The degenerate bright excitons X_B_±__ exhibit initially a strong PL before scattering into hot dark states X_D_. (c and d) In-plane magnetic fields (Voigt geometry; the direction of the **B** field with respect to polarization **P** and propagation direction **k** is shown) split the two bright excitons, polarize them longitudinally (L) and transversely (T) to the field (X_L/T_), and hybridize them with dark and gray states, respectively. Furthermore, magnetic fields switch off specific phonon-driven interband scattering channels, separating excitons into two independent subclasses that emit (c) L- and (d) T-polarized light. This is expected to induce a fast magneto-transport of X_L_ excitons and a strong magneto-PL of X_T_ excitons.

In-plane magnetic fields break the symmetry of the system and split the bright excitons X_B_±__ into two states X_L,T_ that are polarized longitudinally and transversely to the magnetic field, respectively (Figure 1c,d). Magnetic fields have so far been mostly used to probe spins and excitonic properties in perovskites.^27,32–38^ Their impact on exciton emission and diffusion has remained largely unexplored. In this work, we bridge this critical knowledge gap by developing a material-specific and fully predictive theoretical approach supported by temperature-resolved PL experiments. We investigate the phonon-driven exciton dynamics and transport, explicitly incorporating the full exciton fine structure of the prototypical layered (PEA)_2_PbI_4_ perovskite. We theoretically predict and experimentally confirm the transition between bright and dark excitons (Figure 1a,b), leaving characteristic signatures in the time- and temperature-dependent PL. This transition is predicted to occur on a time scale of tens of picoseconds at cryogenic temperatures and is driven by the phonon-mediated interband scattering. Furthermore, we reveal how in-plane magnetic fields drastically enhance exciton diffusion while simultaneously weakening the initial emission of X_L_ excitons (Figure 1c). This is a direct consequence of efficient scattering into dark states and the formation of highly mobile hot dark excitons. In contrast, we show the opposite behavior for X_T_ excitons (i.e., stronger emission and weaker diffusion; Figure 1d), whose interband scattering into dark states is strongly suppressed. Overall, our microscopic study sheds light on the impact of in-plane magnetic fields on exciton emission and diffusion in 2D perovskites.

## Microscopic Model

We first determine the excitonic energy landscape in the investigated prototypical 2D perovskite, (PEA)_2_PbI_4_.^29,30^ We solve the Wannier equation using the Keldysh potential^39–41^ with effective electron and hole masses taken from first-principle calculations.^42^ In this way, we obtain microscopic access to excitonic binding energies (of approximately 220 meV) and excitonic wave functions. Then, we include the exchange interaction and spin–orbit coupling, leading to the exciton fine structure that is of key importance for the phenomena discussed in this work^25–27,30,43–50^ (Figure 1a). We find a dark exciton X_D_ lying approximately 22 meV below two degenerate bright states X_B_±__, which are located approximately 1 meV below gray state X_Z_. Note that deviations of the lattice arrangements from a perfect orthorhombic phase can lead to an anisotropic exchange interaction, giving rise to a small splitting between the two bright states even at zero magnetic field.^30^ Because our focus lies on magneto-optics, where the degeneracy of bright states is lifted, the anisotropic exchange is expected to only lead to a small quantitative correction of the presented results.

Next, we include exciton–phonon scattering within the second-order Born–Markov approximation; see the Supporting Information (SI) for more details.^51^ The momentum-integrated scattering rate with the phonon mode α from the bright to dark state Γ_BD_^α^ shows a negligible contribution from optical phonons at low temperatures (α = op). The absorption of optical phonons is thermally suppressed, while their emission is prohibited by a relaxation bottleneck^30^ because the energy of the most relevant optical phonon is assumed to be 35 meV^41,42^ and thus larger than the bright–dark exciton separation of 22 meV. As a consequence, only long-wavelength acoustic phonons drive the bright-to-dark exciton scattering at small temperatures (α = ac). This leads to a drastically slower formation of dark excitons in the range of tens of picoseconds in (PEA)_2_PbI_4_ compared to tungsten-based TMDs.^51,52^

The application of a magnetic field in the Voigt geometry leads to hybridization of X_L_ and X_D_ excitons as well as X_T_ and X_Z_ excitons, respectively. This results in a transfer of the oscillator strength from bright to dark and gray states, allowing them to emit L- or T-polarized light. Most importantly, we find that the field-induced hybridization strongly restricts the phonon-mediated interband scattering channels within the exciton fine structure, allowing scattering only between X_L_ and X_D_ states and between X_T_ and X_Z_ states, respectively (Figure 1c,d). This creates two independent exciton subclasses *i* with longitudinal (*i* = ∥ with the bands μ_*i*_ = L, D) and transverse (*i* = ⊥ with the bands μ_*i*_ = T, Z) polarization with respect to the magnetic field^30,50^ (Figure 1c,d). This gives rise to remarkable polarization-dependent magneto-optical and magneto-transport phenomena.

Next, we microscopically investigate the spatiotemporal exciton dynamics and explicitly include the excitonic fine structure and phonon-driven scattering channels between the dark, bright, and gray states. To this purpose, we calculate the spatiotemporal dynamics of the excitonic Wigner function,^53,54^ and introducing its spatial average (see the SI), we obtain a generalized Fick’s law for the exciton spatial density *N*_*i*_(**r**,*t*) in the subclass *i*:

1 $${Ṅ}_{i}\left(r,t\right)=D_{i}\left(t\right)\;\Delta_{r}N_{i}\left(r,t\right)-\frac{N_{i}\left(r,t\right)}{\tau_{i}^{\mathrm{rad}}\left(t\right)}$$

Here, 1/τ_*i*_^rad^(*t*) = ∑_*p*_1/τ_*i*,*p*_^rad^(*t*) is the total effective radiative rate, which sums over the emission of photons with the polarization *p* = L, T, Z and is determined by the time-dependent occupation in the lightcone. The first term in eq 1 describes the spatial broadening with the solution $N_{i}\left(r,t\right)=\frac{n_{i}\left(t\right)}{2\pi \sigma_{i}{\left(t\right)}^{2}}e^{-r^{2}/2\sigma_{i}{\left(t\right)}^{2}}$ including the total occupation *n*_*i*_(*t*) and the squared width σ_*i*_(*t*)^2^, which evolves from the initial confinement σ_*i*_(0) via the dynamics σ̇_*i*_^2^(*t*) = 2*D*_*i*_(*t*). The generalized diffusion coefficient *D*_*i*_(*t*)­

2 $$D_{i}\left(t\right)=\frac{1}{n_{i}\left(t\right)}\underset{\mu_{i},Q}{\sum }\tau_{\mu_{i},Q}\frac{\hbar^{2}Q^{2}}{2M^{2}}\rho_{\mu_{i},Q}\left(t\right)$$

with *M* being the total exciton mass and τ_μ_*i*_,**Q**_ the scattering time of states μ_*i*_ with center-of-mass momentum **Q**, goes beyond the conventional diffusion^53,54^ by including transient effects. These are found to introduce the time-resolved spatially averaged exciton distribution ρ_μ_*i*_,**Q**_(*t*); see the SI.

Finally, we calculate the photoluminescence (PL), which corresponds to the radiative loss of excitons in eq 1. The polarization-, space-, and time-resolved PL can be obtained via energy integration of the well-known Elliot formula, yielding

3 $$I_{i,p}\left(r,t\right)=\frac{N_{i}\left(r,t\right)}{\tau_{i,p}^{\mathrm{rad}}\left(t\right)}$$

­(see the SI for more details). The light emission depends on the spatial exciton distribution *N*_*i*_(**r**,*t*) and the polarization-resolved effective radiative time τ_*i*,*p*_^rad^(*t*) with *p* = L, T for *i* = ∥, ⊥ as we focus on normal-incidence emission. Overall, the solution of the coupled eqs 1–3 allows spatial and temporal resolution of the polarization-dependent PL including the excitonic fine structure and the impact of in-plane magnetic fields. The developed theoretical model will be exploited to study bright–dark exciton thermalization and its impact on exciton emission and diffusion with and without magnetic fields. Our microscopic model is material-specific and predictive, offering guidance for the observation of experimentally accessible temperature- and polarization-dependent exciton PL and diffusion.

## Efficient Transient Exciton Emission

We investigate the time-resolved PL in the 2D (PEA)_2_PbI_4_ perovskite after resonant excitation (Figure 2a). At large times, we find that the PL intensity increases with the temperature. This is characteristic of dark materials whose ground state is a dark exciton.^55,56^ Here, the increased thermal population of energetically higher-lying bright states enhances the emission. This is further confirmed by the increased PL emission as a function of temperature at *t* = 100 ps shown in Figure 2b. Interestingly, we find exactly the opposite behavior in the first 25 ps: Here, the emission is stronger at smaller temperatures (see also the orange line in Figure 2b). This behavior can be explained by the weak phonon-driven interband scattering from bright to dark excitons; i.e., the material emits photons within a relatively long time window despite the dark ground state. This can be traced back to a pronounced relaxation bottleneck.^30^ At low temperatures, bright-to-dark scattering is driven by long-wavelength acoustic phonons, whose efficiency decreases linearly toward smaller temperature, reaching a very small value of 0.03 meV at 10 K—almost 2 orders of magnitude smaller than that in tungsten-based TMDs, where the dark ground state becomes populated on a femtosecond time scale.^51,52,57–59^ In 2D perovskites, we find an interband scattering time of ℏ/Γ_BD_ ≈ 20 ps reflecting the temporal evolution of the PL intensity shown in Figure 2. Because only a few dark excitons are formed in the first few tens of picoseconds, (PEA)_2_PbI_4_ behaves like a bright material for the first 25 ps.

**Figure 2:**
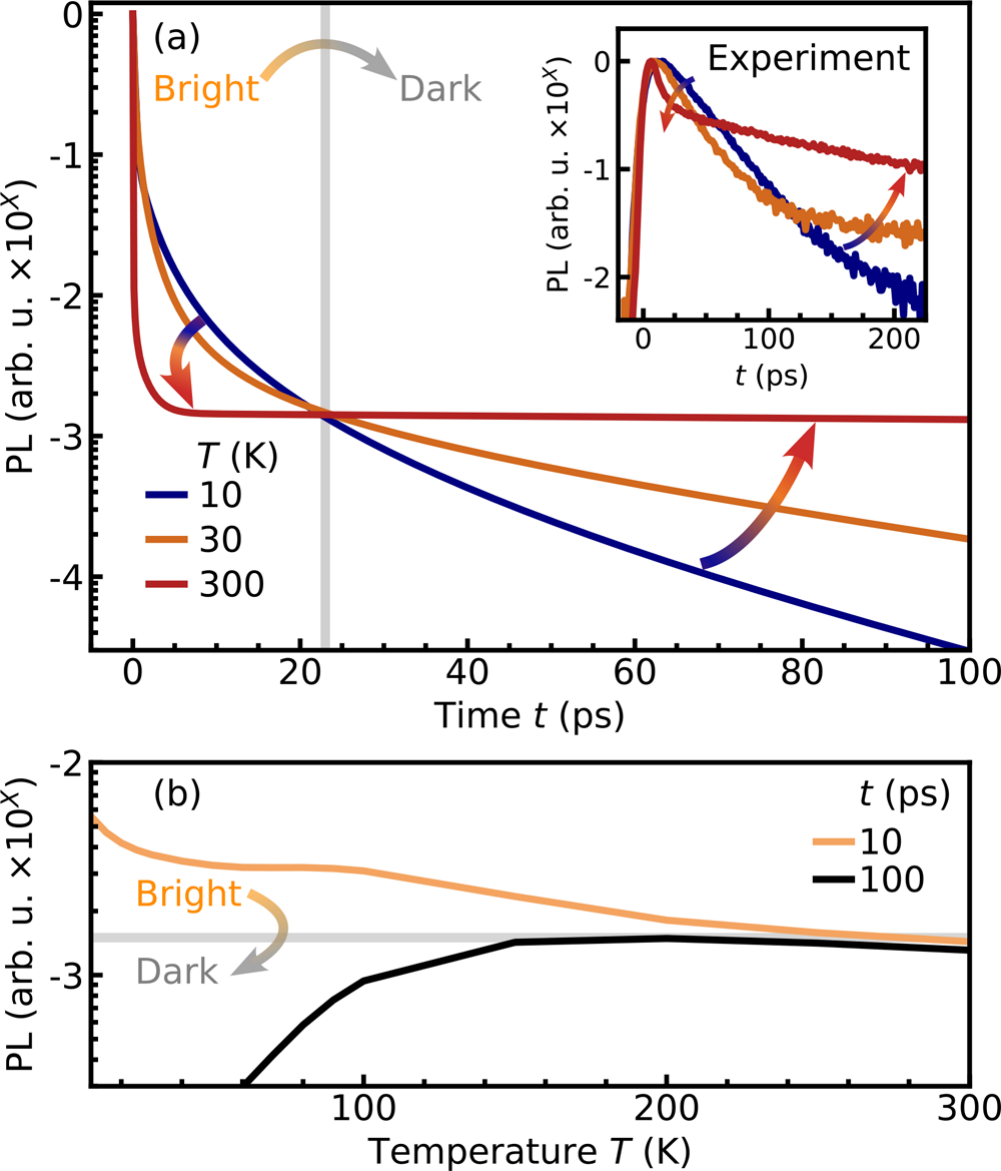
Transient exciton emission. (a) Time-resolved and (b) temperature-resolved PL after resonant excitation for three fixed temperatures and times, respectively. At large times, the PL intensity increases with the temperature, as expected for dark materials. However, before a critical time of about 25 ps, the PL is more efficient at smaller temperatures, reflecting the suppressed formation of dark excitons due to a relaxation bottleneck. This prediction is confirmed by time- and temperature-resolved PL experiments; see the inset.

Interestingly, the delayed occupation of dark states also leads to a quasi-biexponential decay of the PL intensity similar to TMD monolayers:^56^ A first sharp decay occurs while the resonantly excited bright excitons relax, and then the system reaches equilibrium, with the majority of excitons being in the dark state. The resonant excitation triggers an initial intense emission, followed by a fast decay governed by both radiative recombination and exciton–phonon scattering. The latter drives excitons out of the lightcone, resulting in a weakening of the PL emission. At higher temperatures, exciton–phonon scattering is more efficient, resulting in a faster decay. At room temperature, the PL decreases by 3 orders of magnitude in the first few picoseconds, while remaining almost constant in the next 200 ps (decay time of approximately 1 ns). This slower decay takes place once excitons have thermalized and is driven by an effective radiative time τ^rad^ determined by the equilibrium occupation in the lightcone.

The theoretically predicted bright-to-dark exciton transition is confirmed by time- and temperature-resolved PL experiments, cf. the inset of Figure 2a. Here, transient PL microscopy measurements are performed under resonant excitation conditions. A pulsed laser is tuned to the absorption resonance, exciting the sample at 2.36 eV at 5 K and avoiding overlap with the emitted PL signal via a tunable short-pass filter; cf. the SI. As the temperature increases, the excitation energy is shifted accordingly to track the resonance moving to higher energies. The excitation fluence is kept at 3 nJ/cm^2^ over the studied temperature range, well within the linear excitation regime to exclude contributions from nonlinear processes, such as exciton–exciton annihilation.^60,61^ We find signatures of biexponential decay at all measured temperatures, with an initial rapid decay of the exciton population, followed by decelerated recombination dynamics. As the temperature increases, the decay of the first component becomes faster, resulting in an accelerated onset of the slower recombination regime. The opposite trend is visible at later times, where the decay is found to slow down as the temperature rises. This observation is in good qualitative agreement with the theoretical predictions. Compared to the theory, we find a quantitatively slower initial decay of tens of picoseconds, which could be due to the multilayered character of the investigated perovskite samples, where, e.g., reabsorption of photons emitted by another layer could occur.

## Magneto-optics

In-plane magnetic fields separate exciton states into two subclasses with opposite polarization, which show enhanced or suppressed interband scattering between bright and dark states (Figure 1c,d). As a direct consequence, magnetic fields can be used as a tuning knob to change the polarization-dependent PL. Figure 3 shows the modeled time- and polarization-resolved magneto-PL with a moderate magnetic field of *B* = 5 T (in direct comparison to *B* = 0 T). The case of larger magnetic fields *B* is reported in the SI. The PL includes the emission from the two bright states as well as from the dark and gray states, which acquire an oscillator strength via hybridization with the bright states induced by the in-plane magnetic field.^27,30,50^ We find that the T-polarized emission is much stronger than the emission without a magnetic field, as shown by the red line in Figure 3. This can be traced back to the suppressed interband scattering of X_T_ excitons in the dark states. Hence, their occupation persists longer than that for the degenerate X_B_±__ excitons in the absence of a magnetic field, resulting in much more efficient emission. The emission of T-polarized excitons at *B* = 5 T becomes 2–3 orders of magnitude larger than the PL at *B* = 0 T after approximately 100 ps, while remaining almost constant with increasing *B* (cf. the SI). Note that also the gray state X_Z_ acquires an oscillator strength due to field-induced hybridization with X_T_ excitons; however, its emission is negligible because both the oscillator strength and occupation are lower compared to X_T_ excitons. The slow decay of the PL at later times reflects the reduced occupation of the lightcone during the intraband thermalization process.

**Figure 3:**
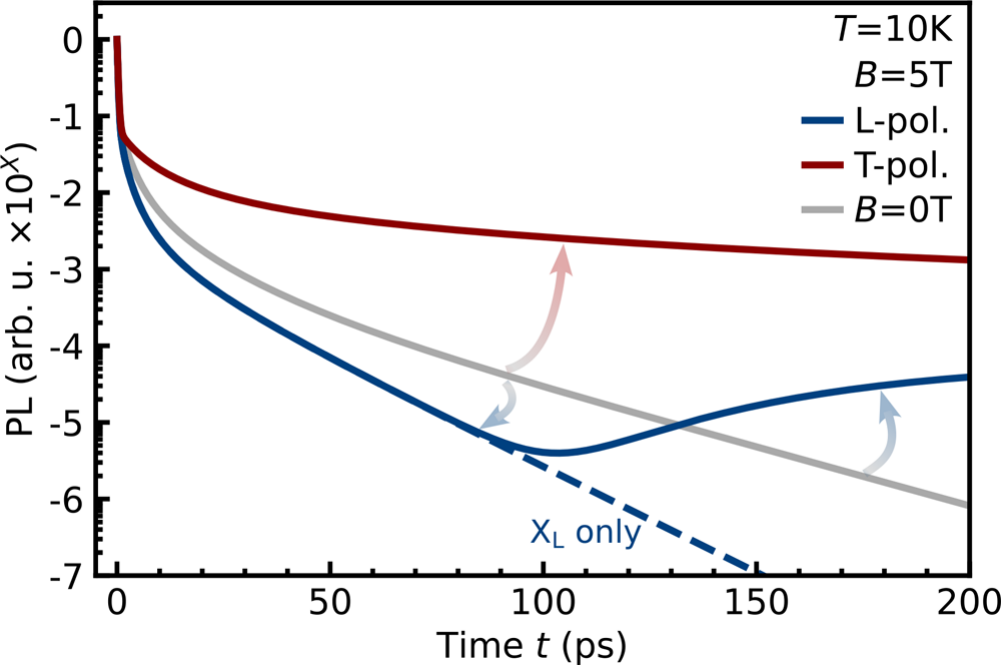
Polarization-dependent magneto-optics. Time-resolved PL at *T* = 10 K with and without a magnetic field (*B* = 5 T). In-plane fields increase the magneto-PL from T-polarized excitons by suppressing scattering into the dark ground states, while the opposite takes place for the emission of L-polarized excitons in the first 100 ps. Here, the PL is initially dominated by X_L_ excitons (denoted by the dashed line), until dark excitons relax into the lightcone and start emitting light due to field-induced hybridization with bright excitons.

In contrast, we find that the emission from L-polarized excitons is weaker than the emission without a magnetic field in the first 100 ps; see the blue line in Figure 3. The X_L_ excitons experience phonon-driven interband scattering two times faster than the degenerate bright excitons X_B_±__ at *B* = 0; cf. the SI. Interestingly, the L-polarized emission shows a nonmonotonic behavior: it starts to increase after approximately 100 ps and even exceeds the emission without a magnetic field. This occurs via the field-induced activation of dark excitons X_D_, which acquire an oscillator strength.^26,27,30^ To demonstrate this, we show the PL emitted solely from X_L_ excitons without the contribution of dark states; see the dashed line in Figure 3. The PL is initially dominated by optically excited bright states. The subsequent interband scattering forms hot dark excitons, which lose their excess energy via quasi-elastic intraband scattering. Once dark excitons relax into the lightcone, the emission from X_D_ excitons becomes much stronger, reflecting their large occupation at 10 K. As further shown in the SI, the emission from X_D_ becomes even more intense at higher magnetic fields, where the hybridization with bright states is even more efficient.

## Exciton Magneto-transport

Now, we investigate the impact of interband exciton thermalization on the magneto-transport of excitons in 2D perovskites. We first consider an initial resonant excitation in the presence of a magnetic field (*B* = 5 T). Unpolarized light excites both T- and L-polarized excitons, which evolve independently and can be addressed individually by polarization of the corresponding PL. In Figure 4a, we show the spatiotemporal evolution of the L-polarized PL intensity *I*_∥,L_(**r**,*t*) normalized to the intensity at **r** = 0. We observe a fast spatial broadening quantified by the evolution of the squared width, Δσ_∥_^2^ = σ_∥_^2^(*t*) – σ_∥_^2^(0), shown in Figure 4b. While a constant diffusion coefficient would result in a linear increase of Δσ_∥_^2^, the predicted sublinear evolution indicates a temporal change in the diffusion coefficient *D*_∥_(*t*) . The latter is shown in Figure 4c as a function of the temperature and time. We predict extremely high transient diffusion coefficients up to 80 cm^2^/s, well beyond the expected value at equilibrium. This is in good qualitative agreement with the measured transient diffusion of 30 cm^2^/s for *B* = 0 T,^42^ considering that we focus on the time-resolved maximum diffusion, while experiments consider a coarse-grained average over a finite time window.

**Figure 4:**
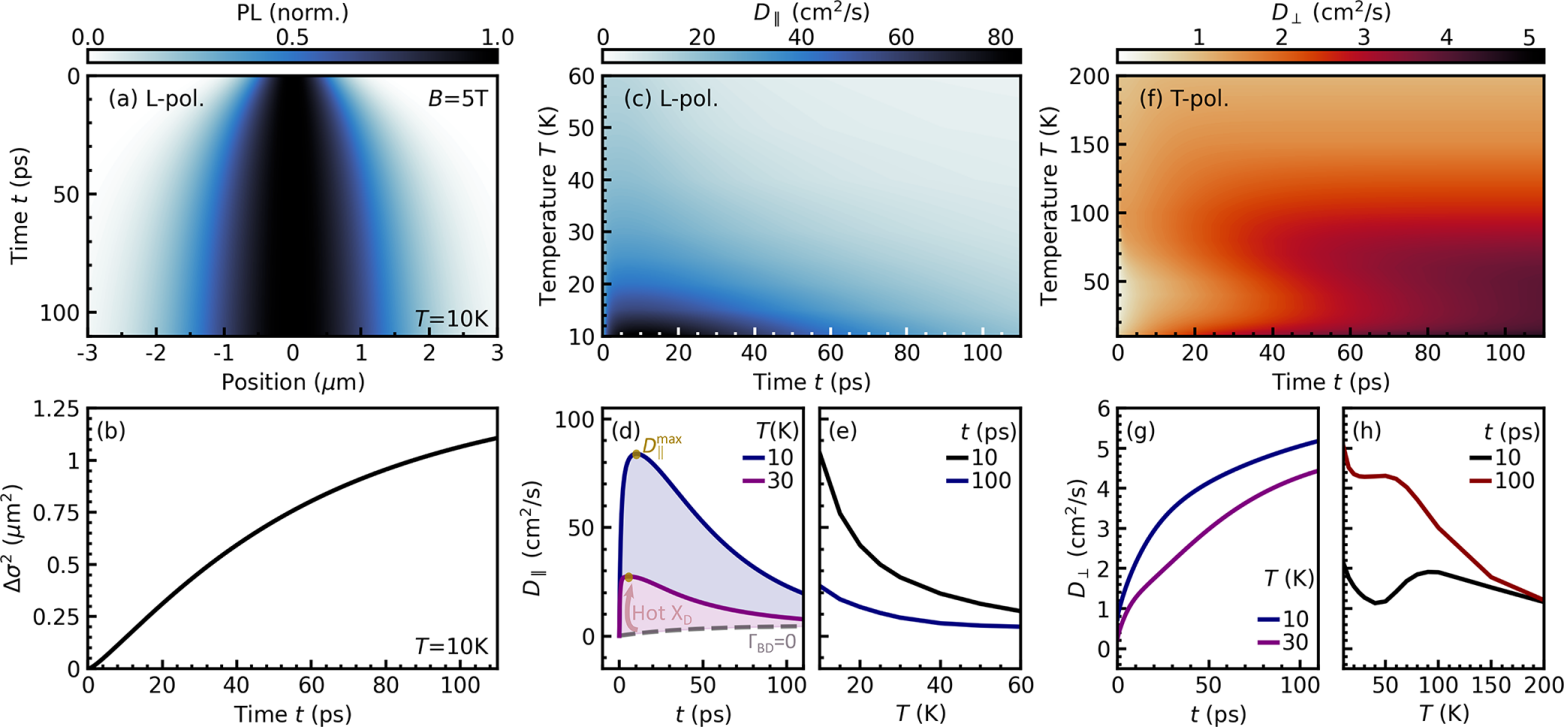
Diffusion of L- and T-polarized excitons. (a) Spatiotemporal PL of L-polarized excitons at *T* = 10 K in the presence of a magnetic field of *B* = 5 T. We find a fast transient diffusion in the first 100 ps, as further illustrated by the (b) temporal evolution of the squared width Δσ_∥_^2^ and (c) transient diffusion coefficient *D*_∥_(*t*) . We predict transient diffusion coefficients of up to 80 cm^2^/s at small temperatures, before a slower quasi-equilibrium diffusion is reached. This is further shown by cuts of the surface plot at fixed (d) temperatures and (e) times. (f–h) Analogous calculations for T-polarized excitons showing no fast diffusion. (g) The initial transient diffusion *D*_⊥_(*t*) is slower than the equilibrium diffusion, and we find (h) an intriguing nonmonotonous temperature dependence.

The transient diffusion coefficient is more than 1 order of magnitude larger than the equilibrium diffusion of approximately 5 cm^2^/s reached after hundreds of picoseconds; see Figure 4d and the SI. The saturation of *D*_∥_(*t*) toward equilibrium is driven by the loss of excess energy of hot dark excitons induced by temperature-dependent intraband scattering. Hence, saturation is reached faster at higher temperatures (Figure 4d). Transient fast diffusion is induced by hot dark excitons formed through quasielastic scattering with acoustic phonons from the bright states (Figure 1c). This is further shown by the drastically reduced diffusion coefficient when the bright-to-dark scattering is artificially switched off (Γ_BD_ = 0) because this results in no hot dark excitons; see the dashed gray line in Figure 4d.

The formation of hot dark excitons can be revealed by tracking the time-resolved and temperature-dependent diffusion coefficient^62^ (Figure 4d,e). The transient diffusion is found to be much faster than the equilibrium diffusion, with the maximum diffusion coefficient *D*_∥_^max^ reached after approximately 10 ps (Figure 4d). Subsequently, the diffusion slows down due to the intraband thermalization with acoustic phonons, resulting in an equilibrium distribution of dark excitons (Figure 1b). Furthermore, the transient diffusion coefficient roughly shows a 1/*T* dependence for relatively small temperatures *T* (blue line in Figure 4e). This is due to the competition of the temperature-dependent scattering time and temperature-independent excess energy and can be considered as a hallmark for the presence of hot dark excitons; see the SI.

The effect of a finite magnetic field *B* ≠ 0 is even more drastic for T-polarized states: In this case, there is no fast transient propagation due to the absence of hot dark excitons, resulting in small diffusion coefficients in the range of just a few cm^2^/s (Figure 4f–h). This can be explained by the field-induced suppression of interband scattering into dark states, directly connected to changes in the transient PL emission. In addition to the large reduction in diffusion, we also find a qualitatively different temporal evolution and temperature dependence of *D*_⊥_(*t*) compared to *D*_∥_(*t*) . Surprisingly, the initial diffusion is now slower than the diffusion at equilibrium (Figure 4g), opposite to the case of L-polarized excitons (Figure 4d). As interband scattering is suppressed, the diffusion is determined by low-mobility excitons close to the lightcone. Once the system is thermalized, the broader equilibrium distribution contains states with higher group velocities. As a result, thermalization accelerates exciton diffusion—similar to the situation in MoSe_2_ monolayers.^62^ Furthermore, we predict an interesting nonmonotonic temperature dependence of the time-resolved diffusion coefficient for T-polarized states (Figure 4f,h). The initial decrease in the diffusion (up to approximately 30 K) is due to the more efficient phonon-mediated scattering that does not get compensated for yet by the thermal energy (the exciton temperature is still lower than the lattice temperature). The subsequent increase reflects the contribution of the quasithermalized higher-mobile excitons outside of the lightcone, followed by another decrease in the diffusion coefficient above 100 K due to activation of the optical phonon scattering channels.

## Magnetic-Field-Dependent Exciton Transport

Now, we investigate the magneto-transport of L- and T-polarized excitons as a function of the field strength *B*. In Figure 5a, we show the maximum diffusion *D*_*i*_^max^ at *T* = 10 K for both L- and T-polarized excitons. Note that, for L polarization, *D*_∥_^max^ corresponds to transient diffusion (Figure 4d), while for T polarization, *D*_⊥_^max^ is found at thermal equilibrium (Figure 4g). Independent of the magnetic field strength, *D*_*i*_^max^ is much higher for L-polarized excitons, reflecting the crucial impact of hot dark excitons on the diffusion. Accordingly, the maximum diffusion scales as 1/*T* for all *B* values; cf. Figure 5b. Importantly, at each temperature, the diffusion is enhanced for increasing magnetic fields, reaching almost 150 cm^2^/s at *B* = 50 T. This can be traced back to the field-induced variation of the exciton wave function and hybridization of the bright and dark/gray states. At *B* = 0 T, the diffusion is polarization-independent and is determined by the scattering channels of the degenerate *B*_±_ state. Note that our theoretical approach does not capture well the transition between the situation at *B* = 0 T and finite magnetic fields because we treat the interband scattering involving the spin-mixed conduction band in a simplified way (cf. eq S6 in the SI). The change in the polarization-dependent diffusion is expected to be continuous due to a smooth field-dependent closing of scattering channels, and thus we restrict our study on the polarization-dependent diffusion to *B* ≥ 5 T (Figure 5a).

**Figure 5:**
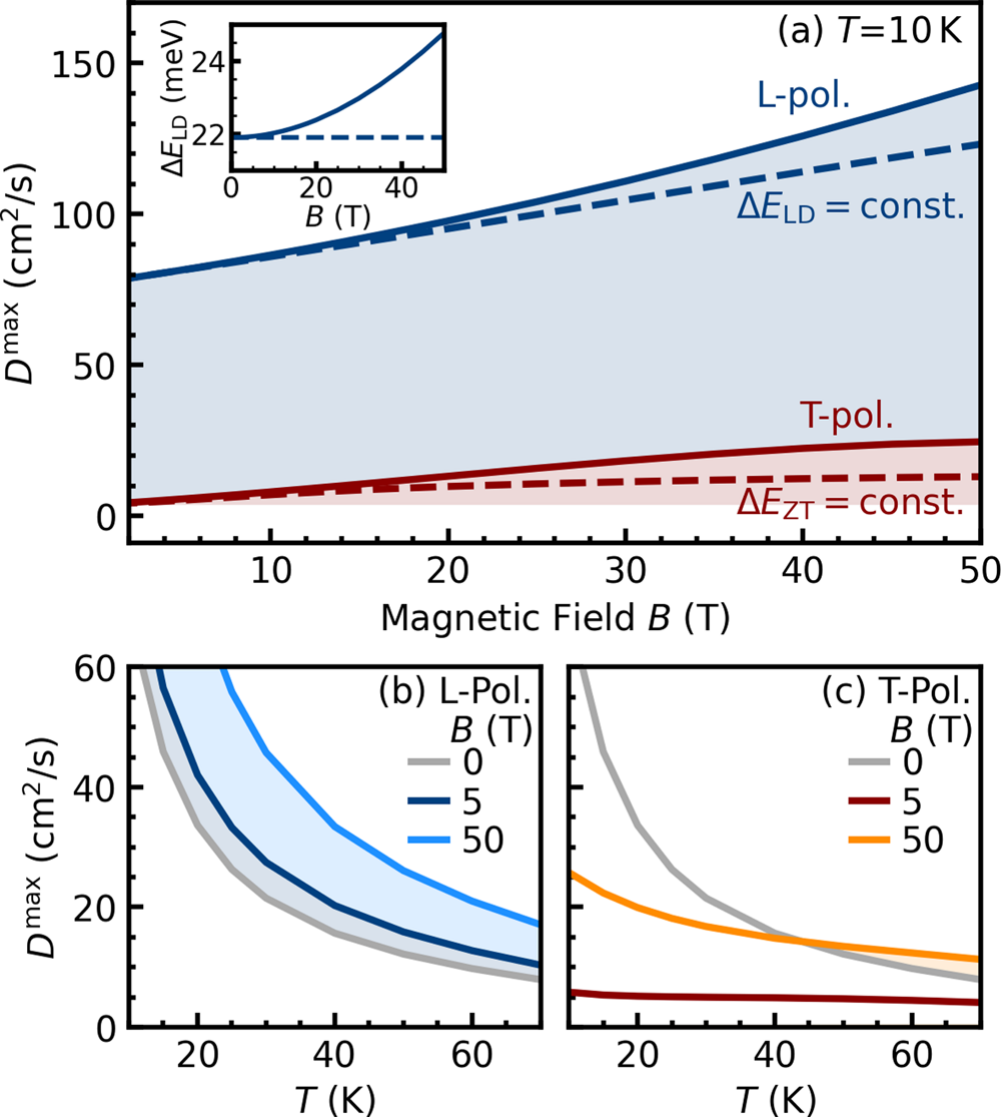
Magneto-transport of excitons. (a) Maximum diffusion for L- and T-polarized excitons as a function of a finite magnetic field *B* at *T* = 10 K. The dashed lines describe the artificial case, where the bright–dark (Δ*E*_LD_) and gray–bright splittings (Δ*E*_ZT_) have been kept constant. The inset illustrates the increase in Δ*E*_LD_ with the magnetic field. The maximum diffusion of X_L_ excitons is driven by hot dark excitons, as revealed by the (b) 1/*T* behavior of *D*_∥_^max^ at all *B*. (c) Even a moderate magnetic field suppresses the formation of hot excitons for X_T_ states, whose diffusion becomes very small and nearly temperature-independent at *B* = 5 T.

In the presence of a magnetic field, the scattering channels between different exciton subclasses *i* = ∥, ⊥ remain closed via a cancellation effect between spin-dependent wave function components with opposite sign.^30^ However, the strength of the allowed scattering channels within each subclass changes with magnetic field *B*. In particular, the intraband scattering rates become weaker for the lower state within each exciton subclass, i.e., for X_D_ and X_T_ excitons. Because the diffusion is dominated by the lowest most populated state, these changes lead to a field-induced enhancement of the maximum diffusion. In particular, the intraband scattering rates for dark excitons decrease quasilinearly with *B* (Figure S1b), resulting in a clearly enhanced *D*_∥_^max^ (Figure 5a).

In addition, field-induced hybridization leads to an avoided crossing, resulting in a red-shift of X_D_ and X_T_ excitons, while X_L_ and X_Z_ states shift blue. As a consequence, the bright–dark energy separation Δ*E*_LD_ = *E*_L,0_ – *E*_D,0_, and hence the hot-exciton excess energy increases quasiquadratically with the field *B*; see the inset of Figure 5a. This results in a nearly quadratic increase in *D*_∥_^max^ with the magnetic field. To demonstrate the importance of the field-induced change in the dark–bright splitting upon exciton diffusion, we artificially keep this splitting constant and consider only the field-induced variation of phonon-mediated scattering rates; see the dashed line in Figure 5a.

In the case of T-polarized states, the maximum diffusion *D*_⊥_^max^ is overall much smaller than that for L-polarized excitons due to the suppressed scattering to dark excitons. Remarkably, the maximum diffusion of T-polarized excitons increases with *B* by almost a factor of 5, from *D*_⊥_^max^ ≈ 5 cm^2^/s at *B* = 5 T up to almost 25 cm^2^/s at *B* = 50 T. This originates from field-induced hybridization of the X_T_ and X_Z_ states. Here, the change of *D*_⊥_^max^ is mostly driven by the field-induced decrease of the intraband scattering rates of X_T_ excitons. Furthermore, the temperature dependence of *D*_⊥_^max^ deviates drastically from the 1/*T* behavior observed for L-polarized excitons (Figure 5c). In this temperature range, *D*_⊥_^max^ depends weakly on the temperature through the competition between different bands (see the SI).

## Conclusions

On the basis of a microscopic, material-specific, and predictive approach, we study the spatiotemporal exciton dynamics in (PEA)_2_PbI_4_ as a prototypical example for 2D perovskites. We predict that PL shows the temperature-dependent behavior of a bright material in the first tens of picoseconds, despite the dark ground state. This is confirmed by time- and temperature-resolved PL experiments and is explained by the appearance of a pronounced relaxation bottleneck, which drastically slows down phonon-driven interband scattering into dark states. Furthermore, we show that the emission and diffusion of excitons can be controlled by an in-plane magnetic field. We predict a considerable field-induced enhancement of the emission of T-polarized excitons due to suppressed scattering into dark states. Furthermore, we find a remarkably fast magneto-transport of L-polarized excitons driven by the formation of highly mobile hot dark excitons, reaching values of 80 cm^2^/s at *T* = 10 K at a relatively weak magnetic field of *B* = 5 T. The gained microscopic insights into the spatiotemporal exciton dynamics subject to magnetic fields provide guidance for experimental studies on excitonic magneto-transport in 2D perovskites.

## Supplementary Material



## Notes

Details on the derivation of the main equations and additional discussion of the magnetic-field-dependent exciton optics and transport (PDF)
