# Supplementary material for: Microscopic Insights into Magneto-optics and Magneto-transport in 2D Perovskites
Source: Nano Lett. 2026 Apr 16;26(16):5468–76. doi: 10.1021/acs.nanolett.6c00182 (PMC13133913; doi:10.1021/acs.nanolett.6c00182)
Supplement: Supplementary file 1 [file nl6c00182_si_001.pdf]

Supporting Information for
Magneto-Optics and Magneto-Transport in 2D Perovskites

Roberto Rosati,[1,2,*] Jonas K. König,[1,2,*] Sophia Terres,[3] Joshua J. P. Thompson,[4] Michał Baranowski,[5] Paulina Płochocka,[5,6] Alexey Chernikov,[3] and Ermin Malic[1,2]

[1] *Department of Physics, Philipps-Universität Marburg, Renthof 7, D-35032 Marburg, Germany*
[2] *mar.quest | Marburg Center for Quantum Materials and Sustainable Technologies, Hans-Meerwein-Straße 6, D-35032 Marburg, Germany*[†]
[3] *Institute of Applied Physics and Würzburg-Dresden Cluster of Excellence ct.qmat, TUD Dresden University of Technology, Dresden, Germany*
[4] *Department of Materials Science and Metallurgy, University of Cambridge, Cambridge, UK*
[5] *Department of Experimental Physics, Wrocław University of Science and Technology Wybrzeże Wyspiańskiego 27, Wrocław 50-370, Wrocław, Poland*
[6] *Laboratoire National des Champs Magnétiques Intenses, EMFL, CNRS UPR 3228, Université Grenoble Alpes, 38042 Grenoble, France; Laboratoire National des Champs Magnétiques Intenses, EMFL, CNRS UPR 3228, Université Toulouse, Université de Toulouse 3, INSA-T, 31400 Toulouse, France*

1. EXCITON ENERGY LANDSCAPE

We evaluate the exciton energy landscape in the investigated two-dimensional $(PEA)_2PbI_4$ perovskite. We first solve the Wannier equation based on material-specific single-particle masses obtained from first-principle calculations[1] and using the Keldysh potential[2] with dielectric constants of 3.3 and 6.1 for the organic environment and the inorganic perovskite layer[3], respectively. Here, excitons localize in the inorganic perovskite layer without a spatial separation of electrons and holes, contrary to vertical[4–7] or lateral TMD heterostructures[8–10]. We obtain exciton binding energies of approximately 220 meV. We find four degenerate states $|Y_{s_e,s_h;\mathbf{Q}}\rangle \equiv \hat{Y}^{\dagger}_{s_e,s_h;\mathbf{Q}}|0\rangle$ with s_e and s_h indicating the electron and hole spin. Here, $Y^{\dagger}_{s_e,s_h;\mathbf{Q}}$ denotes the corresponding creation operator in second quantization. Each electronic state s_e is provided by a mixture of in-plane and out-of-plane p-like orbitals with opposite spins s_e and $\bar{s}_e$, while holes states s_h are composed only by the s-like orbitals with spin s_h[11,12]. Then we include the exchange interaction reading

$$H_{\text{ex}-\text{X}} = \sum_{\substack{\boldsymbol{k_3 k_q} \\ s_1,s_2,s_3,s_4}} \Big[\sum_{\boldsymbol{G}\neq 0} V_{\boldsymbol{G}} \left(\delta_{\bar{s}_1,s_4}\delta_{\bar{s}_3,s_2}\tilde{\Gamma}^{\text{SR}}_{s_1,s_3}(\boldsymbol{G}) + 4s_1s_3\delta_{s_1,s_4}\delta_{s_3,s_2}\Lambda^{k_3,k_4}_{\text{SR}}(\boldsymbol{G})\right) + V_{\boldsymbol{q}} \left(\delta_{\bar{s}_1,s_4}\delta_{\bar{s}_3,s_2}\tilde{\Gamma}^{\text{LR}}_{s_1,s_3}(\boldsymbol{q}) + 4s_1s_3\delta_{s_1,s_4}\delta_{s_3,s_2}\Lambda^{k_3,k_4}_{\text{LR}}(\boldsymbol{q})\right)\Big] \times \varphi_{\boldsymbol{k_4}+\beta\boldsymbol{q}}\varphi^{*}_{\boldsymbol{k_3}-\alpha\boldsymbol{q}}\hat{Y}^{\dagger}_{s_1,s_4;\boldsymbol{q}}\hat{Y}_{s_3,s_2;\boldsymbol{q}} \quad , \tag{S1}$$

where $V_{\mathbf{q}}$ is the Coulomb potential, φ is the excitonic wavefunction in relative momentum, $s = \pm 1/2$ for spin up/down and $\bar{s} = -s$. The labels LR and SR distinguish between long- and short-range interaction (the latter mediated by long wave-vectors G), while the coefficients $\tilde{\Gamma}$ and Λ involve the interaction between different orbitals[12]. As next, we include the exciton-magnetic field Hamiltonian reading

$$H_{X-\text{Mag}} = \frac{\mu_B B}{2}\sum_{\boldsymbol{q}}\sum_{s_1,s_2,s_3,s_4}\left(g^c_{\text{mag}}\delta_{\bar{s}_1,s_3}\delta_{s_2,s_4} - g^v_{\text{mag}}\delta_{s_1,s_3}\delta_{\bar{s}_2,s_4}\right)\hat{Y}^{\dagger}_{s_1s_4;\boldsymbol{q}}\hat{Y}_{s_3s_2;\boldsymbol{q}} \quad , \tag{S2}$$

with the excitonic g-factors $g_c = 2.9$ and $g_v = -1.1$[13] multiplied by the Bohr magneton μ_B and the strength B of the applied in-plane magnetic field. Such a Hamiltonian has no diagonal values, implying that in-plane magnetic fields in Voigt geometry do not lead to a Zeeman shift[14]. Nevertheless, off-diagonal elements lead to a non-trivial inter-exciton mixing. This together with the exchange-driven mixing provides the final states $\hat{X}^{\dagger}_{\mu,\mathbf{Q}} = \sum_{s_e,s_h} D^{s_e s_h}_{\mu,\mathbf{Q}}\hat{Y}^{\dagger}_{s_e s_h;\mathbf{Q}}$ as a linear combination of the four spin states with μ indicating the band index. Rewriting the Hamiltonians in Eqs. (S1)

---

* These authors contributed equally.
† Email: roberto.rosati@physik.uni-marburg.de

and (S2) in matrix notation, the coefficients $D^{s_e s_h}_{\mu\mathbf{Q}}$ are obtained in the case of a perfect orthorombic lattice solving the following eigenvalue equation[12]

$$\left[\begin{pmatrix} I_Z & 0 & 0 & -I_Z \\ 0 & I_r^{\uparrow\uparrow} & 0 & 0 \\ 0 & 0 & I_r^{\downarrow\downarrow} & 0 \\ -I_Z & 0 & 0 & I_Z \end{pmatrix} + \frac{\mu_B}{2} B \begin{pmatrix} 0 & -g_v & g_c & 0 \\ -g_v & 0 & 0 & g_c \\ g_c & 0 & 0 & -g_v \\ 0 & g_c & -g_v & 0 \end{pmatrix}\right] \begin{pmatrix} D^{\uparrow\uparrow}_{\mu\mathbf{Q}} \\ D^{\uparrow\downarrow}_{\mu\mathbf{Q}} \\ D^{\downarrow\uparrow}_{\mu\mathbf{Q}} \\ D^{\downarrow\downarrow}_{\mu\mathbf{Q}} \end{pmatrix} = E_{\mu\mathbf{Q}} \begin{pmatrix} D^{\uparrow\uparrow}_{\mu\mathbf{Q}} \\ D^{\uparrow\downarrow}_{\mu\mathbf{Q}} \\ D^{\downarrow\uparrow}_{\mu\mathbf{Q}} \\ D^{\downarrow\downarrow}_{\mu\mathbf{Q}}, \end{pmatrix} \quad . \tag{S3}$$

Here, $E_{\mu\mathbf{Q}}$ denotes the final exciton energies, while I_r and I_Z describe respectively the in- and of out-of-plane exchange-induced excitonic terms (provided by a proper combination of the coefficients Λ and $\tilde{\Gamma}$[12]). In the absence of magnetic fields, there are two degenerate bright states $\hat{X}^\dagger_{\mathrm{B}\pm,0}$ with the energy $E_{\mathrm{B}\pm,0} \equiv E_{\mathrm{B},0}$ roughly 22 meV above the dark-state energy $E_{\mathrm{D},0}$ and 1 meV below the gray-state energy $E_{\mathrm{Z},0}$. Note that different phases would modify the mixing of conduction-band orbitals[15,16] and hence the excitonic fine structure[12,17]. Here we focus on the intrinsic behaviour of Wannier excitons in an orthorhombic lattice, while deviations from this rigid structure can result in broad white-light emission and exciton self-trapping[18–22], decreased quantum yield[23], and dynamical disorder[24]. Furthermore, lattice distortions away from the perfect orthorhombic phase can lead to a small bright-dark separation via an asymmetric exchange interaction[17], which would affect our results only quantitatively. An in-plane magnetic field lifts the degeneracy of the two bright states, which acquire an in-plane polarization transverse (T) and longitudinal (L) to the field, $\hat{X}^\dagger_{\mathrm{T}}$ and $\hat{X}^\dagger_{\mathrm{L}}$, respectively[12,13]. Furthermore, in-plane magnetic fields break the symmetry, leading to modified coefficients $D^{s_e s_h}_{\mu,\mathbf{Q}}$ which switch off several interband scattering channels. This leads to the appearance of two independent exciton subclasses i with longitudinal ($i =\parallel$ and $\mu_\parallel$=L,D) and transverse polarization ($i =\perp$ and $\mu_\perp$=T,Z)[12,25]. Interband scattering between states belonging to different subclasses is suppressed. We introduce $E_{i,\pm,\mathbf{Q}}$ describing the highest/lowest energy in each subclass i (i.e. $E_{\parallel,\pm,\mathbf{Q}} = E_{\mathrm{L/D},\mathbf{Q}}$ and $E_{\perp,\pm,\mathbf{Q}} = E_{\perp,\mathrm{Z/T},\mathbf{Q}}$) yielding

$$E_{i,\pm,Q} = E_i^0 \pm \frac{\Delta E_i^0}{2}\sqrt{1+\frac{8\bar{g}_i^2}{\Delta E_i^{02}}} \quad , \tag{S4}$$

where $E_i^0 = \frac{1}{2}(E_{i,+,0} + E_{i,-,0})|_{B=0}$ and $\Delta E_i^0 = E_{i,+,0} - E_{i,-,0}|_{B=0}$ are respectively the average energy and energy separation of the two states in the subclass i for the field-free case, while $\bar{g}_{\parallel/\perp} = \frac{1}{\sqrt{2}}(g_c \mp g_v)\frac{\mu_B B}{2}$ describes the combination of g_c and g_v. Within each subclass, the low-energy state undergoes a field-induced redshift, while the high-energy states gets blue-shifted, in analogy to coherent coupling induced by cavity-[26–28], twist-[29] or strain-induced hybridization[30]. For reasonable magnetic fields, these field-induced energy shifts are small, being of the order of 1 meV for B =20 T[12,13,25]. For the subclass $i =\parallel$, the energy difference between the two hybridized states is $\bar{g}_\parallel \ll \Delta E^0_\parallel \approx 22$ meV, scaling roughly quadratically with the magnetic field B, cf. Eq. (S4) and the inset of Fig. 5 in the main manuscript. In the case of $i =\perp$, it is $\Delta E^0_\perp \approx 1\,\mathrm{meV}$ and the energy splitting is parabolic up to $B \approx 20\,\mathrm{T}$, after which it evolves linearly with B leading to an energy separation $\Delta_{\mathrm{ZT}} = E_{\mathrm{Z},0} - E_{\mathrm{T},0} \approx 6\,\mathrm{meV}$ at $B = 50\,\mathrm{T}$.

2. EXCITON-PHONON SCATTERING

The non-trivial mixing of spins in excitonic wavefunctions leads to a peculiar field-dependent exciton-phonon scattering[12]. Introducing the scattering coefficients $\Gamma^{\alpha;\mu\mu'}_{\mathbf{Q}\mathbf{Q}'}$ from exciton $|\mu,\mathbf{Q}\rangle = X^\dagger_{\mu,\mathbf{Q}}|0\rangle$ to $|\mu',\mathbf{Q}'\rangle$ via interaction with acoustic or optical phonons, respectively α=ac,op, we find in the second-order Born-Markov approximation[31–33]

$$\Gamma^{\alpha;\mu\mu'}_{\boldsymbol{Q}\boldsymbol{Q}'} = \frac{2\pi}{\hbar}\sum_{\pm}\left|\Omega^{\mu\mu'}_{\alpha\boldsymbol{Q}'-\boldsymbol{Q}}\right|^2\left(\frac{1}{2}\pm\frac{1}{2}+n_{\alpha\boldsymbol{Q}'-\boldsymbol{Q}}\right)\delta\left(E_{\mu'\boldsymbol{Q}'} - E_{\mu\boldsymbol{Q}} \pm \hbar\omega^\alpha_{\boldsymbol{Q}'-\boldsymbol{Q}}\right) \quad , \tag{S5}$$

where $\omega^\alpha_{\boldsymbol{Q}'-\boldsymbol{Q}}$ and $n_{\alpha\boldsymbol{Q}'-\boldsymbol{Q}}$ are, respectively, the energy and equilibrium Bose-Einstein distribution of a phonon with the mode α and momentum $\boldsymbol{Q}'-\boldsymbol{Q}$ and where $\pm$ distinguishes between emission and absorption of phonons, respectively. The coefficients of the exciton-phonon Hamiltonian read[12],

$$\Omega^{\mu'\mu}_{\alpha\boldsymbol{Q}} = \sum_{s,s',s''}\left(G^{\alpha,\boldsymbol{q}}_{\mathrm{e}} D^{ss''}_{\mu\boldsymbol{q}} D^{s's''*}_{\mu'\boldsymbol{q}} - \delta_{s,s'} G^{\alpha,\boldsymbol{q}}_{\mathrm{h}} D^{s''s}_{\mu\boldsymbol{q}} D^{s''s*}_{\mu'\boldsymbol{q}}\right) \quad , \tag{S6}$$

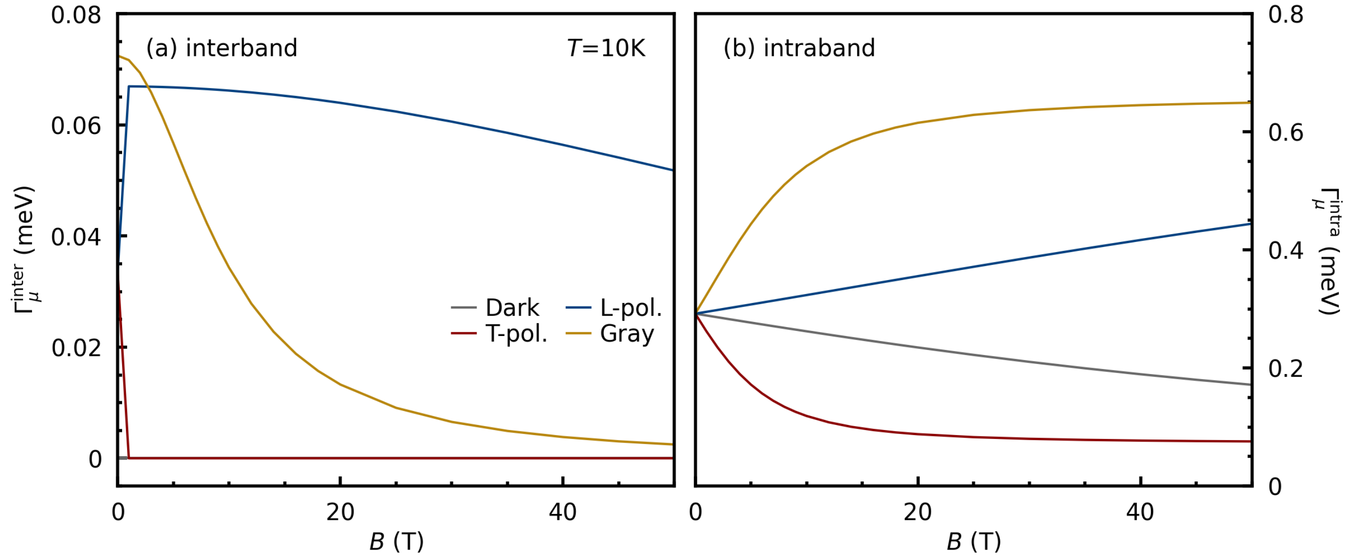

Fig. S1. **Magnetic control of scattering rates** Scattering rates of excitons in the lightcone ($\mathbf{Q}$ =0) for all four bands at $T = 10\,$K distinguishing between (a) inter- and (b) intraband contributions. Due to the optical-phonon bottleneck and the suppressed absorption of optical phonons at cryogenic temperatures, at $B = 0$ the interband scattering from the two degenerate bright states is induced by the quasi-elastic scattering with acoustic phonons into the energetically lower dark state. With a finite magnetic field this channel is abruptly switched off for T-polarized bright states, while it becomes twice stronger for L-polarized excitons. The other inter- and intraband channels show no drastic change as a function of B. Nevertheless, intraband scattering rates smoothly decrease/increase with increasing B for the lower/higher-energy state within each subclass.

with $G_{\rm e}^{\alpha,\boldsymbol{q}} = g^\alpha_{e,\mathbf{q}}\mathcal{F}(\beta\mathbf{q})$ and $G_{\rm h}^{\alpha,\boldsymbol{q}} = g^\alpha_{h,\mathbf{q}}\mathcal{F}(\alpha\mathbf{q})$ depending on the coefficients of the electron- and hole-phonon Hamiltonian, $g^\alpha_{e,\mathbf{q}}$ and $g^\alpha_{h,\mathbf{q}}$, weighted by the excitonic form factor $\mathcal{F}(\boldsymbol{q}) = \sum_{\boldsymbol{k}} \varphi_{\boldsymbol{k}}\varphi^*_{\boldsymbol{k}+\boldsymbol{q}}$, with $\alpha = m_e/(m_e + m_h) = 1 - \beta$ determined by the effective electron and hole masses $m_{e,h}$[12]. The coefficients $g^\alpha_{e,\mathbf{q}}$ and $g^\alpha_{h,\mathbf{q}}$ are obtained from the deformation potential providing temperature-dependent scattering rates and excitonic linewidths[2], hence being all real. From Eq. (S6) we see how electron spin-flipping is in general allowed (by the Elliott-Yaffet mechanism induced by the electronic hybridization in the conduction band[12]), while the hole spin-flipping is always prohibited due to the absence of spin-orbit interaction in the valence band[11,12,34]. Importantly, the coefficients $\Omega^{\mu'\mu}_{\alpha\boldsymbol{Q}}$ depend on the overlap between the wavefunction of the initial and the final exciton state. To visualize this, we write the coefficients $D^{s_e s_h}_{\mu\mathbf{Q}=0} \equiv D^{s_e s_h}_{\mu}$ resolving Eq. (S3) in the presence of a small in-plane magnetic field, which in spinorial notation read[12,25]

$$D_{\rm D} = \frac{1}{\sqrt{2}}(1,0,0,1), \quad D_{\rm T} = \frac{1}{\sqrt{2}}(0,1,-1,0), \quad D_{\rm L} = \frac{1}{\sqrt{2}}(0,1,1,0), \quad D_{\rm Z} = \frac{1}{\sqrt{2}}(1,0,0,-1) \quad . \tag{S7}$$

The two bright states as well as the dark and gray states are composed of analogous spin components $D^{s_e s_h}_{\rm L,T} \equiv D^{s_e s_h}_{\rm L,T}\delta_{s_e,\bar{s}_h}$ and $D^{s_e s_h}_{\rm D,Z} \equiv D^{s_e s_h}_{\rm D,Z}\delta_{s_e,s_h}$. This implies that holes can scatter only intraband, as $\sum_{s,s''} D^{s''s}_{\mu} D^{s''s*}_{\mu'} = \delta_{\mu,\mu'}$. In contrast, electrons can scatter interband via the Elliott-Yaffet mechanism, but with only few channels open, namely the one between the two states composing each subclass. In the absence of any other symmetry-breaking mechanisms, at $B = 0$ the two bright states are fully degenerate, hence they are expected to behave in the same way. In order to describe this with our diagonal density-matrix approach, among the infinite possible representations of these two states we choose $D_{\rm B_+} = (0, 1, 0, 0)$ and $D_{\rm B_-} = (0, 0, 1, 0)$. Similar considerations apply with larger magnetic fields, which maintain similar spinorial structure as with weak fields. For example, one has $D_{\rm D}(B) = (a_{\rm D}, b_{\rm D}, b_{\rm D}, a_{\rm D})$ and $D_{\rm Z}(B) = (a_{\rm Z}, b_{\rm Z}, -b_{\rm Z}, -a_{\rm Z})$, with the component a_μ and b_μ going, respectively, to 1 and 0 for $B \to 0$. This implies that no further opening or closing of scattering channels appears, when increasing the magnetic field B.

Fig. S1 shows the field-dependent inter- and intraband scattering rates at T =10 K. The interband contributions of the two bright states show a drastic change in presence of a small magnetic field: $\Gamma^{\rm inter}_{\rm T}$ goes abruptly to zero, while $\Gamma^{\rm inter}_{\rm L}$ becomes twice larger than without a magnetic field. Both channels are dominated by the quasi-resonant scattering into dark excitons $\rm X_D$ via acoustic phonons, while all other processes are either forbidden by the energy conservation (emission of optical phonons or quasi-resonant scattering into the energetically higher $\rm X_Z$) or negligible by

thermal suppression of the absorption of optical modes at T=10 K. An in-plane magnetic field suppresses the scattering from X_T to X_D, redistributing the scattering efficiency to the rates from X_L to X_D, such that $\Gamma^{\text{inter}}_{B_+} + \Gamma^{\text{inter}}_{B_-} \approx \Gamma^{\text{inter}}_{L} + \Gamma^{\text{inter}}_{T} \approx \Gamma^{\text{inter}}_{L}$. At T = 10K and B = 0, it is $\Gamma^{\text{inter}}_{\text{ac,L}} \approx 0.06$ meV, twice larger than $\Gamma^{\text{inter}}_{B_\pm}$ at B =0 and almost one order of magnitude smaller than the corresponding intervalley contributions of 2-3 meV in WS_2 and WSe_2 monolayers at small temperatures, where interband scattering with finite energy phonons is allowed[35,36]. This leads to a remarkably slower formation of dark excitons in 2D perovskites compared to the femtosecond timescale in TMD mono- and bilayers[37–39]. Note that in real samples, the abrupt field-induced change in the interband scattering rates is expected to be smoother due to possible asymmetries and co-existence of several material phases[12].

While the interband scattering rate of the two bright states changes drastically in presence of a magnetic field, the other interband channels show no drastic change, see Fig. S1(a). At $B = 0$, both X_D and X_Z can interact with both bright states, while at a finite B they can scatter only with X_L and X_T, respectively. However, this is compensated by a two-fold increase of the effectiveness of the open channels, resulting in no abrupt variation of the interband rates $\Gamma^{\text{inter}}_{D/Z}$. Similarly, also the intraband scattering rates show no drastic change as these channels remain always open (Fig. S1(b)). Nevertheless, a magnetic field can vary their magnitude by smoothly modifying the wavefunction spin components. The intraband scattering of X_T decreases by more than a factor of 2 already at $B \approx$10 T, before saturating at a value approximately 4 times smaller than at $B = 0$. Such a decrease is compensated by an increase of the intraband scattering rate of the energetically higher-lying state X_Z in the same subclass. The same behaviour is also found in the other exciton subclass (gray and blue lines in Fig. S1(b)). The decreasing intraband scattering rates of the energetically-lowest state within each subclass results in a speed-up of exciton diffusion with increasing B, as shown in Fig. 5 of the main manuscript and further discussed below in Fig. S2. To see the connection between the exciton-phonon scattering rates and exciton diffusion we now derive the generalized Fick's law and the equation for the transient diffusion coefficient.

3. GENERALIZED FICK'S LAW

To obtain a generalized Fick's law we use the Wigner function[40] $f_{\mu_i}(\mathbf{r}, \mathbf{Q}, t) = \sum_{\mathbf{Q}'} \langle \hat{X}^\dagger_{\mu_i,\mathbf{Q}+\mathbf{Q}'/2} \hat{X}_{\mu_i,\mathbf{Q}-\mathbf{Q}'/2} \rangle e^{\imath \mathbf{Q}'\cdot\mathbf{r}}$ for excitons in band μ_i within the subclass i, with $\mathbf{r}$ and $\mathbf{Q}$ indicating, respectively, center-of-mass position and momentum. Then the exciton density reads $N_i(\mathbf{r}, t) = \frac{1}{\mathcal{V}} \sum_{\mu_i,\mathbf{Q}} f_{\mu_i}(\mathbf{r}, \mathbf{Q}, t)$, where $\mathcal{V}$ is the volume of the system. . Using the Heisenberg equation of motion[41] and the second-order Born-Markov approximation, the spatiotemporal dynamics of the Wigner function can be described by the Boltzmann transport equation[42]

$$\dot{f}_{\mu_i}(\mathbf{r}, \mathbf{Q}, t) = -\mathbf{v}_\mathbf{Q} \cdot \nabla f_{\mu_i}(\mathbf{r}, \mathbf{Q}, t) + \dot{f}_{\mu_i,sc}(\mathbf{r}, \mathbf{Q}, t) + \dot{f}_{\mu_i,r}(\mathbf{r}, \mathbf{Q}, t) \quad . \tag{S8}$$

The first term describes the regular exciton propagation driven by the gradient in the occupation and the group velocity $\mathbf{v}_\mathbf{Q} = \hbar\mathbf{Q}/M$, which in the specific case of the 2D perovskite $(PEA)_2PbI_4$ is independent of the band index μ because all excitons have the same total mass M. The second term describes the scattering with phonons, which can be written as the local Boltzmann collision rate[33,42]

$$\dot{f}_{\mu_i,sc}(\mathbf{r}, \mathbf{Q}, t) = \Gamma^{\text{in}}_{\mu_i,\mathbf{Q}}(\mathbf{r}, t) - \Gamma^{\text{out}}_{\mu_i,\mathbf{Q}} f_{\mu_i}(\mathbf{r}, \mathbf{Q}, t) \quad , \tag{S9}$$

where $\Gamma^{\text{in}}_{\mu_i,\mathbf{Q}}(\mathbf{r}, t) = \frac{1}{\hbar} \sum_{\alpha,\mu'_i,\mathbf{Q}'} \Gamma^{\alpha;\mu'_i\mu_i}_{\mathbf{Q}'\mathbf{Q}} f_{\mu'_i}(\mathbf{r}, \mathbf{Q}', t)$ provides the local in-scattering term and $\Gamma^{\text{out}}_{\mu_i,\mathbf{Q}} = \frac{1}{\hbar} \sum_{\alpha,\mu'_i,\mathbf{Q}'} \Gamma^{\alpha;\mu_i\mu'_i}_{\mathbf{Q}\mathbf{Q}'}$ the corresponding out-scattering rate, which is related to the state-dependent scattering time $\tau_{\mu_i,\mathbf{Q}}$ via $\tau_{\mu_i,\mathbf{Q}} = 1/\Gamma^{\text{out}}_{\mu_i,\mathbf{Q}}$. Finally, the third term in Eq. (S9) describes the radiative recombination

$$\dot{f}_{\mu_i,r}(\mathbf{r}, \mathbf{Q}, t) = -\tilde{\Gamma}^{\text{rad}}_{\mu_i,\mathbf{Q}} f_{\mu_i}(\mathbf{r}, \mathbf{Q}, t) \quad , \tag{S10}$$

where we have introduce the radiative recombination rate $\tilde{\Gamma}^{\text{rad}}_{\mu_i,\mathbf{Q}} = \frac{1}{\hbar} \sum_p \Gamma^{\text{rad}}_{\mu_i,p,\mathbf{Q}}$ as a sum over the emitted photon polarization p=L,T,Z. The latter depends on the band- and polarization-dependent oscillator strength in the lightcone $\Gamma^{\text{rad}}_{\mu,p,\mathbf{Q}} = \Gamma^{\text{rad}}_{\mu,p,0} \Theta\left[q_\mu - |\mathbf{Q}|\right]$ with $q_\mu = \frac{E_{\mu,\mathbf{Q}=0}}{\hbar c}$ being the lightcone edge, while $\Gamma^{\text{rad}}_{\mu,p,0}$ describes the band-dependent oscillator strength. Starting with a field- and polarization-independent overall rate $\Gamma^{\text{rad}}_0 = \sum_{\mu_i} \Gamma^{\text{rad}}_{\mu_i,p,0} = 2.66$ meV^2, the magnetic field- and band-dependent radiative rate $\tilde{\Gamma}^{\text{rad}}_{\mu_i,0}$ is obtained from the wavefunctions in Eq. (S3)[25]. This results in a transfer of the oscillator strength from the two bright states toward the dark and gray state, which become optically activated and can interact with normal-incidence photons.

We now investigate the spatio-temporal dynamics of the spatial exciton density $N_i(\mathbf{r},t)$,

$$\dot{N}_i(\mathbf{r},t) = \frac{1}{\mathcal{V}}\sum_{\mu_i,\mathbf{Q}} \dot{f}_{\mu_i}(\mathbf{r},\mathbf{Q},t) \quad . \tag{S11}$$

At large times the Wigner function is typically in the local quasi-equilibrium, i.e., $f_{\mu_i}(\mathbf{r},\mathbf{Q},t) \approx f^{\circ}_{\mu_i}(\mathbf{r},\mathbf{Q},t) = \mathcal{V}N_i(\mathbf{r},t)\rho^{\circ}_{\mu_i,\mathbf{Q}}/n_i(t)$, where $n_i(t) = \int d\mathbf{r}\ N_i(\mathbf{r},t)$ is the total exciton occupation and $\rho^{\circ}_{\mu_i,\mathbf{Q}}$ is the equilibrium distribution. Including this quasi-equilibrium approximation to the Wigner transport equation leads to the conventional Fick's law[40,42,43]. However, since the equilibrium distribution is time-independent, this method cannot capture the transient diffusion occurring, while excitons relax in energy. In this work, we present an extension capable of capturing the diffusion dynamics prior to the onset of thermal equilibrium. For this purpose, we rewrite the Wigner function as

$$f_{\mu_i}(\mathbf{r},\mathbf{Q},t) = \frac{\mathcal{V}}{n_i(t)}N_i(\mathbf{r},t)\rho_{\mu_i,\mathbf{Q}}(t) + \delta f_{\mu_i}(\mathbf{r},\mathbf{Q},t) \quad , \tag{S12}$$

where $\rho_{\mu_i,\mathbf{Q}}(t)$ is the spatially averaged exciton distribution,

$$\rho_{\mu_i,\mathbf{Q}}(t) = \frac{1}{\mathcal{V}}\int d\mathbf{r} f_{\mu_i}(\mathbf{r},\mathbf{Q},t) \quad , \tag{S13}$$

while $n_i(t)$ is the total exciton occupation, which can be now written also as $n_i(t) = \sum_{\mu_i,\mathbf{Q}} \rho_{\mu_i,\mathbf{Q}}(t)$. Here $\delta f_{\mu_i}(\mathbf{r},\mathbf{Q},t)$ provides the local deviation of the Wigner function from its spatial average

$$\delta f_{\mu_i}(\mathbf{r},\mathbf{Q},t) = f_{\mu_i}(\mathbf{r},\mathbf{Q},t) - \frac{\mathcal{V}}{n_i(t)}N_i(\mathbf{r},t)\rho_{\mu_i,\mathbf{Q}}(t) \quad . \tag{S14}$$

From Eq. (S14) it follows $\sum_{\mu_i,\mathbf{Q}} \delta f_{\mu_i}(\mathbf{r},\mathbf{Q},t) = 0$ for every point $\mathbf{r}$ and time t. Inserting Eq. (S12) into Eqs. (S9) and (S11) the dynamics of $N_i(\mathbf{r},t)$ reads

$$\dot{N}_i(\mathbf{r},t) \approx \frac{1}{\mathcal{V}}\sum_{\mu_i,\mathbf{Q}}\left[-\mathbf{v}_{\mathbf{Q}}\cdot\nabla_{\mathbf{r}}\delta f_{\mu_i}(\mathbf{r},\mathbf{Q},t) - \frac{\mathcal{V}}{n_i(t)}N_i(\mathbf{r},t)\tilde{\Gamma}^{\text{rad}}_{\mu,p,\mathbf{Q}}\rho_{\mu_i,\mathbf{Q}}(t)\right] \quad , \tag{S15}$$

where all other terms vanish either due to particle conservation of scattering processes or are approximated to zero by assuming that the spatially averaged exciton distribution is isotropic in momentum, implying $\rho_{\mu_i,\mathbf{Q}}(t) \approx \rho_{\mu_i,-\mathbf{Q}}(t)$, and $\sum_{\mu_i,\mathbf{Q}} g_{\mathbf{Q}}\delta f(\mathbf{r},\mathbf{Q},t) \approx 0$ for all smooth non-oscillating functions $g_{\mathbf{Q}}$, since $\delta f(\mathbf{r},\mathbf{Q},t)$ oscillates between positive and negative values. While the radiative rates $\Gamma^{\text{rad}}_{\mu,p,\mathbf{Q}}$ vary abruptly in momentum as $\Theta\left[q_\mu - |\mathbf{Q}|\right]$, we assume that the Wigner function deviates in the lightcone only weakly from the averaged value, namely $\delta f_{\mu_i}(\mathbf{r},\mathbf{Q} \approx 0,t) \ll \frac{\mathcal{V}}{n_i(t)}N_i(\mathbf{r},t)\rho_{\mu_i,\mathbf{Q}\approx 0}(t)$. Similar approximations allow also to rewrite the scattering-induced dynamics in Eq. (S9) to

$$\dot{f}_{\mu_i,sc}(\mathbf{r},\mathbf{Q},t) \approx -\Gamma^{\text{out}}_{\mu_i,\mathbf{Q}}\delta f_{\mu_i}(\mathbf{r},\mathbf{Q},t) + \frac{\mathcal{V}}{n_i(t)}N_i(\mathbf{r})\dot{\rho}_{\mu_i,\mathbf{Q},sc}(t) \quad , \tag{S16}$$

where

$$\dot{\rho}_{\mu_i,\mathbf{Q},sc}(t) = \Gamma^{\text{in}}_{\mu_i,\mathbf{Q}}(t) - \Gamma^{\text{out}}_{\mu_i,\mathbf{Q}}\rho_{\mu_i,\mathbf{Q}}(t) \tag{S17}$$

with $\Gamma^{\text{in}}_{\mu_i,\mathbf{Q}}(t) = \frac{1}{\hbar}\sum_{\alpha,\mu_i',\mathbf{Q}'}\Gamma^{\alpha;\mu'\mu}_{\mathbf{Q}'\mathbf{Q}}\rho_{\mu_i',\mathbf{Q}'}(t)$ as the in-scattering term of the spatially averaged distribution. While obtained from the local scattering-induced dynamics of the Wigner function in Eq. (S9), the results in Eq. (S17) provide the typical Boltzmann scattering dynamics for the space-independent momentum distribution $\rho_{\mu_i,\mathbf{Q}}(t)$. Using Eq. (S16), Eq. (S8) and $\tau_{\mu_i,\mathbf{Q}} = 1/\Gamma^{\text{in}}_{\mu_i,\mathbf{Q}}$ we obtain

$$\delta f_{\mu_i}(\mathbf{r},\mathbf{Q},t) \approx -\tau_{\mu,\mathbf{Q}}\left[\dot{f}_{\mu_i}(\mathbf{r},\mathbf{Q},t) + \mathbf{v}_{\mathbf{Q}}\cdot\nabla f_{\mu_i}(\mathbf{r},\mathbf{Q},t) - \dot{f}_{\mu_i,r}(\mathbf{r},\mathbf{Q},t) - \frac{\mathcal{V}}{n_i(t)}N_i(\mathbf{r})\dot{\rho}_{\mu_i,\mathbf{Q}}(t)\right] \quad . \tag{S18}$$

Finally, we insert Eq. (S18) into Eq. (S15) and we make the crucial dynamic quasi-average approximation, where we assume that $\delta f_i^{\mu}(\mathbf{r},\mathbf{Q},t)$ is small enough that we can approximate the Wigner function with its spatial average

yielding

$$f_{\mu_i}(\mathbf{r},\mathbf{Q},t) \approx \frac{\mathcal{V}}{n_i(t)}\mathcal{V}N_i(\mathbf{r},t)\rho_{\mu_i,\mathbf{Q}}(t) \quad . \tag{S19}$$

Furthermore, exploiting again the isotropy of $\rho_{\mu_i,\mathbf{Q}}(t)$ as well as of $\dot{\rho}_{\mu_i,\mathbf{Q}}(t)$ and $\dot{f}^{\mu}_{i,r}(\mathbf{r},\mathbf{Q},t)$ we find

$$\dot{N}_i(\mathbf{r},t) \approx D_i(t)\Delta_{\mathbf{r}}N_i(\mathbf{r},t) - \frac{N_i(\mathbf{r},t)}{\tau_i^{\mathrm{rad}}(t)} \quad , \tag{S20}$$

with

$$D_i(t) = \frac{1}{n_i(t)}\sum_{\mu_i,\mathbf{Q}} \tau_{\mu_i,\mathbf{Q}}\frac{\hbar^2Q^2}{2M^2}\rho_{\mu_i,\mathbf{Q}}(t) \quad , \tag{S21a}$$

$$\frac{1}{\tau_i^{\mathrm{rad}}(t)} = \sum_p \frac{1}{\tau_{i,p}^{\mathrm{rad}}(t)} = \frac{1}{n_i(t)}\sum_{\mu_i,\mathbf{Q}}\tilde{\Gamma}^{\mathrm{rad}}_{\mu_i,\mathbf{Q}}\rho_{\mu_i,\mathbf{Q}}(t) \quad . \tag{S21b}$$

The result of Eqs. (S20) closely resembles the conventional Fick's law of diffusion, however, with the difference of the time-dependent effective diffusion coefficient and the effective radiative time defined in Eqs. (S21a) and (S21b). Their evolution is dictated by the spatially averaged distribution $\rho_{\mu_i,\mathbf{Q}}(t)$, whose dynamics according to Eq. (S13) is derived from the dynamics of $f_{\mu_i}(\mathbf{r},\mathbf{Q},t)$ and according to Eqs. (S17) and (S10) reads

$$\dot{\rho}_{\mu_i,\mathbf{Q}}(t) = \frac{1}{\mathcal{V}}\int d\mathbf{r}\dot{f}_{\mu_i}(\mathbf{r},\mathbf{Q},t) = \dot{\rho}_{\mu_i,\mathbf{Q},sc}(t) - \tilde{\Gamma}^{\mathrm{rad}}_{\mu_i,\mathbf{Q}}\rho_{\mu_i,\mathbf{Q}}(t) \quad , \tag{S22}$$

where we have used Eq. (S12) and where all terms involving $\delta f_{\mu_i}(\mathbf{r},\mathbf{Q},t)$ are neglected in view of the spatial integration. Importantly, the scattering with phonons as well as the radiation of photons affect both Eqs. (S20) and (S22), with the last terms in Eq. (S22) providing a depopulation of the lightcone states (compared to states just outside the lightcone) due to their radiative recombination.

We stress that introducing the normalized spatially averaged exciton distribution $\bar{\rho}_{\mu_i,\mathbf{Q}}(t) = \rho_{\mu_i,\mathbf{Q}}(t)/n_i(t)$, we can again recover Eq. (S20), while rewriting Eqs. (S21) and (S22) as

$$D_i(t) = \sum_{\mu_i,\mathbf{Q}} \tau_{\mu,\mathbf{Q}}\frac{\hbar^2Q^2}{2M^2}\bar{\rho}_{\mu_i,\mathbf{Q}}(t) \quad , \tag{S23a}$$

$$\frac{1}{\tau_i^{\mathrm{rad}}(t)} = \sum_p \frac{1}{\tau_{i,p}^{\mathrm{rad}}(t)} = \sum_{\mu_i,\mathbf{Q}}\tilde{\Gamma}^{\mathrm{rad}}_{\mu_i,\mathbf{Q}}\bar{\rho}_{\mu_i,\mathbf{Q}}(t) \quad , \tag{S23b}$$

and

$$\begin{aligned}\dot{\bar{\rho}}_{\mu_i,\mathbf{Q}}(t) =& \frac{\partial}{\partial t}\frac{\int d\mathbf{r} f_{\mu_i}(\mathbf{r},\mathbf{Q},t)}{\sum_{\mu_i,\mathbf{Q}}\int d\mathbf{r} f_{\mu_i}(\mathbf{r},\mathbf{Q},t)} = \\ =& \dot{\bar{\rho}}_{\mu_i,\mathbf{Q},sc}(t) - \tilde{\Gamma}^{\mathrm{rad}}_{\mu_i,\mathbf{Q}}\bar{\rho}_{\mu_i,\mathbf{Q}}(t) - \bar{\rho}_{\mu_i,\mathbf{Q}}(t)\frac{\dot{n}_i(t)}{n_i(t)} \quad .\end{aligned} \tag{S24}$$

Here the last term preserves the normalization of $\bar{\rho}_{\mu_i,\mathbf{Q}}(t)$ despite the decrease of the total occupation $n_i(t)$, whose dynamics reads

$$\dot{n}_i = \int d\mathbf{r}\frac{1}{\mathcal{V}}\sum_{\mu_i,\mathbf{Q}}\dot{f}_{\mu_i,r}(\mathbf{r},\mathbf{Q},t) = -\frac{n_i(t)}{\tau_i^{\mathrm{rad}}(t)} \quad . \tag{S25}$$

The last two terms of Eq. (S24) induce a redistribution out of the lightcone in the normalized distribution $\bar{\rho}_{\mu_i,\mathbf{Q}}(t)$, which can lead to a deviation of the equilibrium distribution[28], which in turn is the equilibrium solution of $\dot{\bar{\rho}}_{\mu_i,\mathbf{Q},sc}(t) = 0$.

In a nutshell, the derivation presented in this section allows to generalize the conventional Fick's law obtained at equilibrium[40,42] to the case of transient non-equilibrium diffusion. Hence, the approach allows us to capture the transient fast diffusion induced by hot dark excitons.

4. ELLIOT FORMULA

An effective radiative time appears in Eqs. (S21b) and (S23b). Here, we explore its impact on the photon dynamics. We first introduce the annihilation (creation) operators $c_{\mathbf{q}}^{p(\dagger)}$ for photons with the polarization p, momentum $\mathbf{q}$ and energy $\mathcal{E}_{p,\mathbf{q}} = \hbar c\sqrt{k_z^2+k^2}$ with k_z being the out-of-plane component of the photon. The temporal evolution of the photonic occupation density $\rho_{\mathbf{q}}^{c,p} = \langle c_{\mathbf{q}}^{p\dagger} c_{\mathbf{q}}^{p}\rangle$ describes the photoluminescence. The dynamics of $\rho_{\mathbf{q}}^{c,p}$ can be described by the Elliot formula as[36]

$$\dot{\rho}_{\mathbf{q}}^{c,p} = \frac{1}{\hbar}\sum_{\mu,\mathbf{q}} \left|M_{\mathbf{q}}^{p}\right|^2 \frac{\left(\Gamma_{\mu,p,\mathbf{q}}^{\mathrm{rad}} + \hbar\Gamma_{\mu,\mathbf{q}}^{\mathrm{out}}\right)\rho_{\mu,\mathbf{Q}}}{\left(E_{\mu,\mathbf{q}} - \mathcal{E}_{p,\mathbf{q}}\right)^2 + \frac{1}{4}\left(\Gamma_{\mu,p,\mathbf{q}}^{\mathrm{rad}} + \hbar\Gamma_{\mu,\mathbf{q}}^{\mathrm{out}}\right)^2} \quad , \tag{S26}$$

where for the sake of simplicity we have omitted the argument t and the subclass index i. Here, $\left|M_{\mathbf{q}}^{p}\right|^2$ is the excitonic optical dipole moment, which is related to the radiative dephasing $\Gamma_{\mu,p,\mathbf{q}}^{\mathrm{rad}}/2$ via[36]

$$\Gamma_{\mu,p,\mathbf{q}}^{\mathrm{rad}} = 2\pi \sum_{\mathbf{k}} \left|M_{p\mathbf{k}}^{\mu}\right|^2 \delta\left(E_{\mu,\mathbf{q}} - \mathcal{E}_{p,\mathbf{q}+\mathbf{k}}\right) \quad . \tag{S27}$$

The energy conservation in Eq. (S27) implies that $\Gamma_{\mu,p,\mathbf{q}}^{\mathrm{rad}}$ vanishes for all momenta larger than the lightcone $q > q_\mu$. The exciton dispersion is almost constant within the lightcone, i.e. $E_{\mu,\mathbf{q}} \approx E_{\mu,0}$ for $q < q_\mu$. In this approximation, assuming the rotational isotropy of all quantities and rewriting the summation in Eq. (S27) as an integral in energy with $kdk = \mathcal{E}d\mathcal{E}/(\hbar^2c^2)$, we can resolve the integral and invert Eq. (S27) to write the $\mathbf{q}$-independent optical matrix element in the lightcone as $\left|M_{p\mathbf{q}}^{\mu}\right|^2 = \Gamma_{\mu,p,\mathbf{q}}^{\mathrm{rad}} \frac{\hbar^2c^2}{\mathcal{V}E_{\mu,0}}$, i.e., we can rewrite the matrix element in terms of the radiative rate. Including this in Eq. (S26), converting again the sum over momentum to an integral in energy under the approximation $E_{\mu,\mathbf{q}} \gg \Gamma_{\mu,p,\mathbf{q}}^{\mathrm{rad}} + \hbar\Gamma_{\mu,\mathbf{q}}^{\mathrm{out}}$, the energy- and space-integrated photoluminescence $I_{i,p}$ reads

$$I_{i,p} = \sum_{\mathbf{q}} \dot{\rho}_{\mathbf{q}}^{c,p} = \frac{n_i}{\tau_{i,p}^{\mathrm{rad}}} \quad , \tag{S28}$$

which as expected from particle conservation corresponds to the decay of $n_i(t)$, cf. Eq. (S25). Finally, for broad exciton profiles we can generalize Eq. (S28) to describe the local emission as $I_{i,p}(\mathbf{r}) = \frac{N_i(\mathbf{r})}{\tau_{i,p}^{\mathrm{rad}}}$.

5. EQUILIBRIUM MAGNETO-DIFFUSION

In the main part of the manuscript we have mostly focused on the transient diffusion $D_i(t)$ because this is remarkably fast for L-polarized excitons. Here, we focus on the equilibrium diffusion D_i° appearing after exciton thermalization. This is particularly interesting for T-polarized excitons ($i = \perp$) because the maximum diffusion coefficient $D_{\perp}^{\mathrm{max}}$ is typically reached at equilibrium, cf. Figs. 4(g) of the main manuscript. Only at very high field strengths we find a small non-thermal transient diffusion induced by the slightly hot excitons formed in the $\mathrm{X_T}$ band via scattering from $\mathrm{X_Z}$ excitons, as the energy separation increases to $\Delta E_{\mathrm{ZT}} \approx 6\,\mathrm{meV}$ allowing slightly larger excess energies. Such a transient diffusion is, however, only partially faster (max 25% at $T = 10\,\mathrm{K}$ and $B = 50\,\mathrm{T}$, where $D_{\perp}^{\mathrm{max}} \approx 1.25 D_{\perp}^{\circ}$), hence it is not shown here, where we rather focus on the equilibrium diffusion.

In Fig. S2 we show the temperature-dependent equilibrium diffusion D_i° for both T- and L-polarized excitons. We consider two different in-plane magnetic fields and observe that at each temperature or polarization D_i° increases with increasing B. This occurs due to the field-induced weakening of the intraband scattering rates of $\mathrm{X_T}$ and $\mathrm{X_D}$ excitons, as shown in Fig. S1(b). These two bands are the energetically lowest and hence the most occupied states in the corresponding exciton subclass $i = \perp, \parallel$. A field-induced increase of their scattering time $\tau_{\mathrm{D/T},\mathbf{Q}}$ results in an overall increase of D_i° with B for each temperature and polarization, cf. Eq. (S21a). Now we focus on the temperature variation of D_i° for a given field strength B. In the moderate-/high-temperature regime $T \gtrsim 50$ K, increasing temperature results in a drop of D_i° for both polarizations. This is induced by the thermal activation of the absorption of optical phonons, which induce an abrupt increase of scattering rates and hence a decrease of diffusion[44]. Surprisingly, the two subclasses show a different temperature-dependent equilibrium diffusion at small temperatures $T \lesssim 50$K. L-polarized excitons show an almost temperature-independent diffusion coefficient. This is a typical behaviour in quasi-classical transport[45,46], as the linear increase of thermal energy is compensated by a linear decrease of scattering times, which at low temperatures T are dominated by the interaction with acoustic phonons.

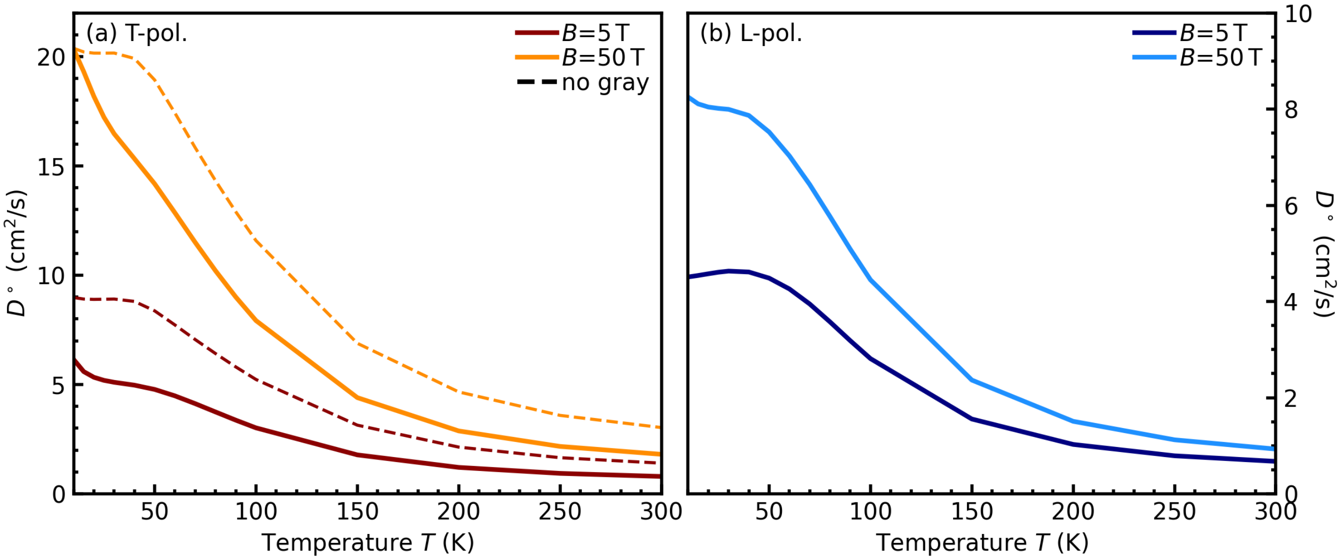

Fig. S 2. **Equilibrium diffusion.** Temperature-dependent equilibrium diffusion of (a) T- and (b) L-polarized excitons for different magnetic fields. At each temperature and polarization the equilibrium diffusion increases with larger magnetic field B due to the field-induced decrease of intraband scattering rates, cf. Fig. S1. The diffusion drops as a function of temperature due to the thermal activation of optical phonons. At small temperatures $T \lesssim 50$K the T-polarized diffusion $D_\perp$ becomes surprisingly smaller with increasing temperature due to the thermal activation of the less-mobile gray states. Removing artificially the gray states results in no decrease for $T \lesssim 50$K, see dashed lines.

In contrast, the equilibrium diffusion $D^\circ_\perp$ shows a surprising decrease at small temperatures, cf. Fig. S2(a). This can be traced back to a competition between the T-polarized bright state X_T and the gray state X_Z. The latter is slower due to the larger number of scattering channels and thus higher scattering rates, cf. Fig. S1.

To better understand this competition, we introduce the band-intrinsic equilibrium diffusion coefficient $D^\circ_{i,\mu_i} = \sum_\mathbf{Q} \tau_{\mu_i,\mathbf{Q}} \frac{\hbar^2 Q^2}{2M^2 n_{\mu_i}} \rho^\circ_{\mu_i,\mathbf{Q}}$ with $n_{\mu_i} = \sum_\mathbf{Q} \rho^\circ_{\mu_i,\mathbf{Q}}$ being the exciton density in band μ_i. At small temperatures, $D^\circ_{i,\mu}$ is almost temperature independent again due to the cancellation between thermal energy and intraband scattering time. The radiative depopulation of the lightcone has a small impact on the diffusion, as excitons with a relative energy much smaller than the thermal one ($\hbar^2 q_\mu^2/2M \ll k_B T$) contribute. However, the band-intrinsic diffusion coefficients $D^\circ_{i,\mu}$ depend on the magnetic field B, as the latter decreases the scattering time of X_Z compared to X_T, resulting in $D^\circ_{\perp,\mathrm{Z}} < D^\circ_{\perp,\mathrm{T}}$. On the other hand, the overall diffusion is an average of the band-intrinsic diffusion constant weighted by the respective occupation[47], $D^\circ_i = \sum_{\mu_i} D^\circ_{i,\mu_i} n^\circ_{\mu_i}/n^\circ_i$ with $n^\circ_i = \sum_{\mu_i} n^\circ_{\mu_i} = 1$. In the specific case of the T-polarized subclass $i = \perp$ this can be rewritten as

$$D^\circ_\perp(T) = D^\circ_{\perp,\mathrm{T}}(T=0) \frac{1 + \frac{D^\circ_{\perp,\mathrm{Z}}(T=0)}{D^\circ_{\perp,\mathrm{T}}(T=0)} e^{-\frac{\Delta E_\mathrm{ZT}(B)}{k_B T}}}{1 + e^{-\frac{\Delta E_\mathrm{ZT}(B)}{k_B T}}} \approx D^\circ_{\perp,\mathrm{T}}(T=0) + \Delta D_\mathrm{ZT}(B) e^{-\frac{\Delta E_\mathrm{ZT}(B)}{k_B T}} \quad , \tag{S29}$$

where we have introduced the field-dependent diffusion difference $\Delta D_\mathrm{ZT}(B) = \left[D^\circ_{\perp,\mathrm{Z}}(T=0) - D^\circ_{\perp,\mathrm{T}}(T=0)\right]$ and energy difference $\Delta E_\mathrm{ZT} = E_{\mathrm{Z},0} - E_{\mathrm{T},0}$. Furthermore, we have used the Boltzmann distribution appearing to state that $n^\circ_{\mu_i}/n^\circ_i = e^{-\frac{\Delta E_{\mu_i \mathrm{T}}(B)}{k_B T}}/(1+e^{-\frac{\Delta E_\mathrm{ZT}(B)}{k_B T}})$, while in the last step we have made the approximation $e^{-\frac{E_{\mathrm{Z},0}-E_{\mathrm{T},0}}{k_B T}} \ll 1$ only for the second term. At finite magnetic fields, the diffusion difference is negative, $\Delta D_\mathrm{ZT}(B) < 0$, resulting in a decrease of $D^\circ_i(T)$ with increasing temperatures, see Fig. S2(a). The strength of the decrease depends exponentially on the energy separation $\Delta E_\mathrm{ZT}(B) > 0$, which for the T-polarized subclass $i = \perp$ is particularly small and comparable to the thermal energy, $\Delta E_\mathrm{ZT}(B) \approx 1$ meV for $B \lesssim 20$ T. Thanks to this small energy difference, the second term becomes relevant. Analogous considerations can be done for the competition between X_D and X_L excitons with $D_\parallel(T)$ described in Eq. (S29) after replacing $\perp, \mathrm{Z}, \mathrm{T} \to \parallel, \mathrm{L}, \mathrm{D}$. In this case, however, $\Delta E_\mathrm{LD}(B) = E_{\mathrm{L},0} - E_{\mathrm{D},0} \approx 22$ meV $\gg k_B T$, resulting in a temperature-independent $D^\circ_\parallel(T) \approx D^\circ_{\parallel,\mathrm{D}}(T=0)$, see Fig. S2(b).

To confirm the role of the competition between X_T and X_Z excitons, in the dashed lines of Fig. S2 we artificially remove the gray states, which as expected results in a temperature-independent $D_\perp$ for $T \lesssim 50$ K (dashed lines). Eq.

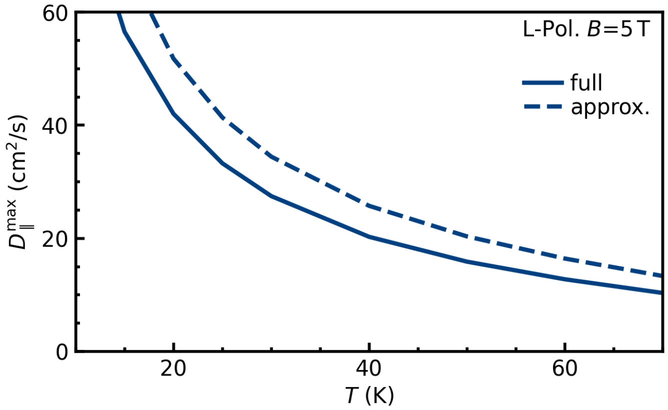

Fig. S 3. **Temperature-dependent hot-exciton diffusion.** Maximum L-polarized diffusion under a moderate magnetic field $B = 5\,\mathrm{T}$ as a function of temperature. The realistic values from the full spatio-temporal dynamics (solid) are compared to the approximated value $D_{\parallel,\mathrm{ap.}}^{\max}$ obtained from Eq. (S30) with the bright-dark energy separation taken as energy average $\langle E\rangle_t \approx \Delta E_{\mathrm{LD}}$. The good agreement confirms that the $1/T$ decrease of the transient diffusion is a hall-mark for hot excitons.

(S29) shows how the decrease of $D_\perp(T)$ with increasing temperature is stronger for larger $|\Delta D_{\mathrm{ZT}}(B)|$ or smaller $\Delta E_{\mathrm{ZT}}(B)$. At $B = 50\,\mathrm{T}$ and T=10 K, the diffusion is unaffected by the gray states because the thermal energy is much smaller than $\Delta E_{\mathrm{ZT}}(B = 50\,\mathrm{T}) \approx$6 meV. Increasing the temperature results in an activation of gray states and hence in the decrease of the diffusion. At smaller B, the energy separation becomes smaller, resulting in a slowed-down diffusion via occupation of the gray states already at $T = 10\,\mathrm{K}$. Here, we stress that already at $B = 5\,\mathrm{T}$ the scattering rates $\Gamma^{\mathrm{intra}}_{\mathrm{T/Z}}$ change, respectively, by a factor of 1/2 and 2 compared to the field-free case, cf. Fig. S1(b). As a consequence, moderate fields are sufficient to strongly change $\Delta D_{\mathrm{ZT}}(B)$, while affecting only weakly $\Delta E_{\mathrm{ZT}}(B)$ due to its quasi-quadratic variation with the field. Furthermore, at large temperatures the diffusion without gray states is faster due to the suppression of the interband scattering from $\mathrm{X_T}$ to $\mathrm{X_Z}$ via absorption of optical phonons, a channel otherwise thermally activated and effective at large temperatures.

In a nutshell, the equilibrium diffusion of T-polarized excitons becomes surprisingly smaller at enhanced temperatures reflecting the competition between between $\mathrm{X_T}$ and $\mathrm{X_Z}$ excitons. This is driven by the small energy separation $\Delta E_{\mathrm{ZT}} \approx$1 meV, contrary to the case of L-polarized excitons, where the much larger energy separation leads to the typical temperature-independent quasi-classical diffusion at small temperatures[44,46].

6. TEMPERATURE-DEPENDENT TRANSIENT DIFFUSION

The transient L-polarized diffusion decreases roughly as $1/T$ for temperatures T in the cryogenic range ($T \lesssim 60 - 70\,\mathrm{K}$), see Figs. 4(d) and 5(b) in the main manuscript. This is a hallmark of the transient formation of hot dark excitons. In contrast, the absence of hot exciton results in an almost temperature-independent T-polarized diffusion, see Fig. 5(c) in the main paper and Fig. S2. To better understand the temperature-dependence of the diffusion coefficient, we come back to Eq. (S21a). The transient diffusion $D_\parallel(t)$ is driven by hot dark excitons, whose scattering time only weakly depends on momentum, as they cannot emit optical phonons, $\tau_{\mathrm{D},\mathbf{Q}} \approx \tau_{\mathrm{D},0} = \tau_{\mathrm{D}}$. Furthermore, dark states are highly populated and thus $n_{\mathrm{D}}(t)$ dominate the sum in Eq. (S21a) as well as the total exciton occupation $n_\parallel(t) \approx n_{\mathrm{D}}(t)$. This allows us to rewrite $D_\parallel(t)$ as a product of τ_{D}/M and the time-dependent average exciton energy $\langle E\rangle_t$ as

$$D_\parallel(t) \approx \frac{\tau_{\mathrm{D}}}{M}\langle E\rangle_t \quad , \tag{S30}$$

with $\langle E\rangle_t = \frac{1}{n_{\mathrm{D}}(t)}\sum_{\mathbf{Q}} \rho_{\mathrm{D},\mathbf{Q}}(t)\frac{\hbar^2 Q^2}{2M}$. At small temperatures, the scattering time of hot dark excitons τ_{D} behaves as $1/T$, as they can interact only with long-wavelength acoustic phonons, i.e., the scattering rate scales linearly with T following the phonon occupation. At equilibrium, the average energy corresponds to the thermal energy $\langle E\rangle_t \approx k_B T$. In contrast, in the transient phase such an average is governed by the lattice-temperature-independent excess energy, $\langle E\rangle_t \approx \Delta E_{\mathrm{LD}}$. As a result, in equilibrium we find a temperature-independent diffusion coefficient $D_\parallel^\circ \approx k_B T \tau_{\mathrm{D}}/M$[40,43,44]. In contrast, the transient diffusion can be described by $D_{\parallel,\mathrm{ap.}}^{\max} \approx \Delta E_{\mathrm{LD}}\tau_{\mathrm{D}}/M$ at first

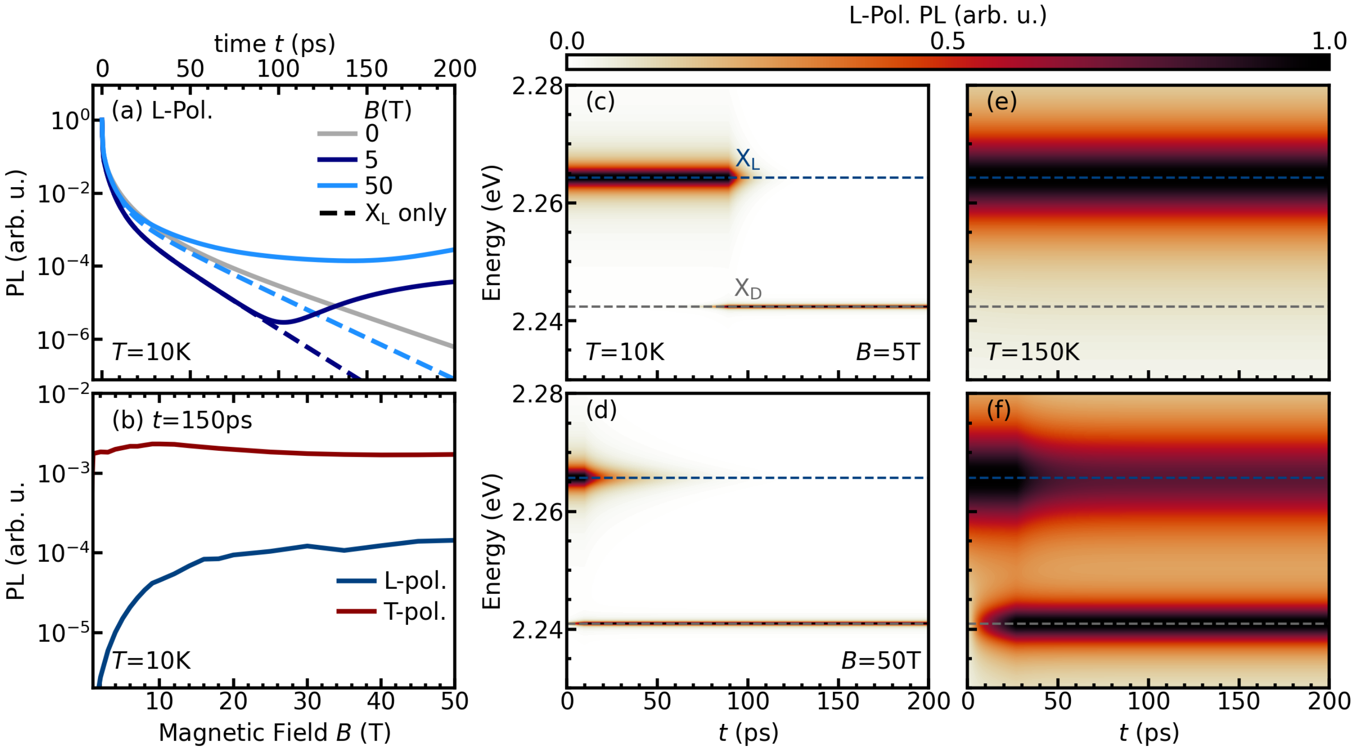

Fig. S4. **Polarization- and temperature-dependent magneto-optics.** Time-resolved photoluminescence at T =10 K for B=0, 5, and 50 T with (a) L- and (b) T-polarized emission, showing a field-induced brightening of the latter. The L-polarized PL shows a delayed emission from the dark state X_D, as further demonstrated in the normalized PL spectrum at B =5 and 50 T (c,d). The emission of X_D is driven by the field-induced optical activation of X_D and becomes relatively weaker at larger temperatures (e, f), where the energetically higher bright states X_L become thermally occupied.

approximation. This is shown in Fig. S3, where we find a very good agreement between the realistic value of $D_\parallel^{\max}$ stemming from the full spatio-temporal dynamics at B=5 T and its approximated value (with microscopic field-dependent scattering time τ_D and bright-dark separation ΔE_{LD}). The approximation slightly overestimates the diffusion because it does not include the effects of intraband thermalization, which induces a loss of excess energy of hot dark excitons during their formation.

7. EXCITON EMISSION AT LARGE MAGNETIC FIELDS

A moderate magnetic field B enhances the T-polarized emission, while weakening the L-polarized PL in the first 100 ps, as shown in Fig. 3 of the main manuscript for B =5T. In Fig. S4 we compare these results to the case of larger fields up to B =50 T. We focus on the time-resolved L-polarized emission, because the T-polarized one behaves qualitatively similar with moderate or large magnetic fields, as we show in Fig. S4(b) by considering the time-resolved PL at time t =150 ps as a function of magnetic field. Here, we see how for each field strength the T-polarized emission is enhanced compared to the L-polarized one, analogously to what seen in the main part of the manuscript for B=5 T. This happens due to the suppressed scattering into the dark states, hence making bright excitons the most occupied states of the T-polarized subclass $i=\perp$. Overall, the T-polarized emission weakly depends on B. This happens because increasing the field strength B the reduced emission from X_T excitons induced by the redistribution of oscillator strength is partially compensated by the increased contribution of X_Z excitons.

As discussed in the main manuscript, the L-polarized emission is initially dominated by the emission from the bright X_L exciton. Subsequently, when hot dark excitons thermalize toward the lightcone of X_D, the low-temperature emission shows a boost induced by the recombination of originally dark X_D excitons. Larger magnetic fields further increase the optical activation of X_D, whose delayed emission hence i) starts earlier and ii) is stronger with larger fields, see Fig. S4(a). At large times, most of the excitons are already in the dark band. Since this gets optically activated by the magnetic field, at t = 150 ps we see a strong enhancement of L-polarized emission with increasing strength B, cf. Fig. S4(b). To better demonstrate this, in Fig. S4(c-f) we explore the time-resolved L-polarized PL

as in Eq. (S26), with the emission normalized at each time t for field strengths B =5, 50 T and for temperatures T=10,150 K. At low temperature, the PL shows a crossover from the high- to the low-energy peak both at small and large magnetic fields, see Figs. S4(c,d). Such a crossover takes place earlier at larger fields, reflecting the stronger field-induced activation of X_D. At both field strengths, the linewidth of the high-energy peak is dominated by the radiative contribution. The low-energy peak, in contrast, is much narrower, as it stems from X_D, whose radiative rates are much smaller than the scattering-induced rate of approximately 0.3 meV (Fig. S1), which hence dominates the linewidth. Despite the much smaller radiative rate, the dark-exciton emission becomes prominent at T=10 K thanks to the much higher occupation. At larger temperatures, this changes thanks to the thermal activation of the energetically higher bright states. At T = 150 K, the high-energy peak emitted from X_L is stronger than the X_D peak at B = 5 T and still comparable to it even at B = 50 T, see Fig. S4(e,f). Here both peaks are much broader that at T=10 K due to the more efficient scattering with phonons. The high-energy peak is broader, since X_L exhibits a larger scattering rate than X_D, see Fig. S1(b).

8. TRANSIENT PHOTOLUMINESCENCE MICROSCOPY

For the experimental measurements of Fig. 2 in the main manuscript, we used a thin-layer sample micromechanically exfoliated from a bulk crystal of (n = 1) layered perovskite phenylethylammonium lead iodide $(PEA)_2PbI_4$. The exfoliated perovskite flake with a thickness on the order of 50 to 70 nm was encapsulated on both sides with tens of nanometers thick layers of hexagonal boron nitride (h-BN) to prevent oxidation and photo-induced degradation. The resulting sample was then mounted in a micro-cryostat for temperature dependent measurements under high vacuum conditions ($< 10^{-6}$ mbar). Transient photoluminescence microscopy measurements were performed at varying temperatures between 5 and 300 K using an 80 MHz pulsed Ti:sapphire laser with a pulse width of 140 fs. To achieve resonant excitation conditions over the entire studied temperature range, we coupled the Ti:sapphire laser into an optical parametric oscillator to obtain photon energies between 2.36 eV at 5 K and 2.41 eV at 300 K. In the excitation path, we used a tunable short pass filter (Semrock VersaChrome EdgeTM, TSP01-561) to spectrally suppress the low-energy flank of the laser, thereby avoiding overlap with the emitted PL signal. The resonant excitation ensures a clean comparison to theoretical predictions that assume near-instantaneous generation of excitons in the bright states. It also allows us to directly exclude any additional sources of overheated and non-equilibrium exciton generation by avoiding any additional excess energy to excitons induced by the initial excitation. Furthermore, the laser was focused to a spot size of about 0.5 μm on the sample using a glass-corrected 60x microscope objective to yield an excitation energy density of 3 nJ/cm^2 per pulse. The residual scattered laser light was spectrally suppressed and separated from the emitted PL signal using a sharp-edge tunable long pass filter (Semrock VersaChrome EdgeTM, TLP01-561). The PL signal was then guided into the spectrometer. A CCD and a streak camera were used for time-integrated and time-resolved measurements, respectively.

Note that exciton dynamics can generally depend strongly on a number of factors including the chemical composition as well as presence of defects and imperfections[48]. These typically impact the recombination dynamics that we observe at longer timescales, limiting the total exciton lifetimes due to the interplay of radiative and non-radiative recombination and exciton trapping. In the presented data in Fig. 2 in the main manuscript, this would affect the comparison between the experimentally measured decay on longer timescales and the theoretical predictions that exclude non-radiative trapping. This leads to shorter exciton lifetimes towards the room temperature conditions in the experiment compared to theory. However, we would like to emphasize that the key reported phenomena is the fast depopulation of bright exciton states with a characteristic temperature dependence. This is demonstrated both in experiment and theory. The agreement is semi-quantitative, since extrinsic processes that have not been taken into account in the microscopic theory can affect the specific decay rates due to defects and imperfections in the studied samples.

REFERENCES

[1] J. D. Ziegler, J. Zipfel, B. Meisinger, M. Menahem, X. Zhu, T. Taniguchi, K. Watanabe, O. Yaffe, D. A. Egger, and A. Chernikov, Nano Letters **20**, 6674 (2020).
[2] D. Feldstein, R. Perea-Causín, S. Wang, M. Dyksik, K. Watanabe, T. Taniguchi, P. Plochocka, and E. Malic, The Journal of Physical Chemistry Letters **11**, 9975 (2020).
[3] X. Hong, T. Ishihara, and A. V. Nurmikko, Phys. Rev. B **45**, 6961 (1992).
[4] P. Rivera, J. R. Schaibley, A. M. Jones, J. S. Ross, S. Wu, G. Aivazian, P. Klement, K. Seyler, G. Clark, N. J. Ghimire, J. Yan, D. G. Mandrus, W. Yao, and X. Xu, Nat. Commun. **6**, 6242 (2015).
[5] N. Leisgang, S. Shree, I. Paradisanos, L. Sponfeldner, C. Robert, D. Lagarde, A. Balocchi, K. Watanabe, T. Taniguchi, X. Marie, R. J. Warburton, I. C. Gerber, and B. Urbaszek, Nat. Nanotechnol. **15**, 901 (2020).

[6] J. Hagel, S. Brem, C. Linderälv, P. Erhart, and E. Malic, Phys. Rev. Res. **3**, 043217 (2021).
[7] J. K. König, J. M. Fitzgerald, J. Hagel, D. Erkensten, and E. Malic, 2D Mater. **10**, 025019 (2023).
[8] R. Rosati, I. Paradisanos, L. Huang, Z. Gan, A. George, K. Watanabe, T. Taniguchi, L. Lombez, P. Renucci, A. Turchanin, B. Urbaszek, and E. Malic, Nat. Commun. **14**, 2438 (2023).
[9] L. Yuan, B. Zheng, Q. Zhao, R. Kempt, T. Brumme, A. B. Kuc, C. Ma, S. Deng, A. Pan, and L. Huang, ACS Nano **17**, 15379 (2023).
[10] E. Vandoolaeghe, F. Fortuna, S. K. Chakraborty, B. Nayak, T. Taniguchi, K. Watanabe, P. K. Sahoo, T. Chervy, and P. A. Murthy, arXiv preprint arXiv:2509.24465 (2025).
[11] M. A. Becker, R. Vaxenburg, G. Nedelcu, P. C. Sercel, A. Shabaev, M. J. Mehl, J. G. Michopoulos, S. G. Lambrakos, N. Bernstein, J. L. Lyons, T. Stöferle, R. F. Mahrt, M. V. Kovalenko, D. J. Norris, G. Rainò, and A. L. Efros, Nature **553**, 189 (2018).
[12] J. J. P. Thompson, M. Dyksik, P. Peksa, K. Posmyk, A. Joki, R. Perea-Causin, P. Erhart, M. Baranowski, M. A. Loi, P. Plochocka, and E. Malic, Adv. En. Mater. **14**, 2304343 (2024).
[13] M. Dyksik, H. Duim, D. K. Maude, M. Baranowski, M. A. Loi, and P. Plochocka, Sci. Adv. **7**, eabk0904 (2021).
[14] S. Raiber, P. E. Faria Junior, D. Falter, S. Feldl, P. Marzena, K. Watanabe, T. Taniguchi, J. Fabian, and C. Schüller, Nat. Commun. **13**, 4997 (2022).
[15] A. D. Wright, C. Verdi, R. L. Milot, G. E. Eperon, M. A. Pérez-Osorio, H. J. Snaith, F. Giustino, M. B. Johnston, and L. M. Herz, Nature communications **7**, 11755 (2016).
[16] E. Fransson, J. Wiktor, and P. Erhart, The Journal of Physical Chemistry C **127**, 13773 (2023).
[17] M. Dyksik, M. Baranowski, J. J. P. Thompson, Z. Yang, M. R. Medina, M. A. Loi, E. Malic, and P. Plochocka, Adv. En. Mater. **15**, 2570044 (2025).
[18] C. M. Mauck and W. A. Tisdale, Trends in Chemistry **1**, 380 (2019).
[19] D. Cortecchia, S. Neutzner, A. R. Srimath Kandada, E. Mosconi, D. Meggiolaro, F. De Angelis, C. Soci, and A. Petrozza, J. Am. Chem. Soc. **139**, 39 (2017).
[20] M. D. Smith, A. Jaffe, E. R. Dohner, A. M. Lindenberg, and H. I. Karunadasa, Chem. Sc. **8**, 4497 (2017).
[21] D. Cortecchia, J. Yin, A. Bruno, S.-Z. A. Lo, G. G. Gurzadyan, S. Mhaisalkar, J.-L. Brédas, and C. Soci, J. Mater. Chem. C **5**, 2771 (2017).
[22] J. Yin, H. Li, D. Cortecchia, C. Soci, and J.-L. Brédas, ACS Ener. Lett. **2**, 417 (2017).
[23] X. Gong, O. Voznyy, A. Jain, W. Liu, R. Sabatini, Z. Piontkowski, G. Walters, G. Bappi, S. Nokhrin, O. Bushuyev, M. Yuan, R. Comin, D. McCamant, S. O. Kelley, and E. H. Sargent, Nat. Mater. **17**, 550 (2018).
[24] J. Nishida, J. P. Breen, K. P. Lindquist, D. Umeyama, H. I. Karunadasa, and M. D. Fayer, J. Am. Chem. Soc. **140**, 9882 (2018).
[25] J. K. König, J. M. Fitzgerald, and E. Malic, Nano Lett. **25**, 8519 (2025).
[26] H. Deng, H. Haug, and Y. Yamamoto, Rev. Mod. Phys. **82**, 1489 (2010).
[27] C. Schneider, M. M. Glazov, T. Korn, S. Höfling, and B. Urbaszek, Nat. Commun. **9**, 2695 (2018).
[28] J. M. Fitzgerald, R. Rosati, B. Ferreira, H. Shan, C. Schneider, and E. Malic, Optica **11**, 1346 (2024).
[29] S. Brem, C. Linderälv, P. Erhart, and E. Malic, Nano Lett. **20**, 8534 (2020).
[30] A. M. Kumar, D. Yagodkin, R. Rosati, D. J. Bock, C. Schattauer, S. Tobisch, J. Hagel, B. Höfer, J. N. Kirchhof, P. Hernández López, K. Burfeindt, S. Heeg, C. Gahl, F. Libisch, E. Malic, and K. I. Bolotin, Nat. Commun. **15**, 7546 (2024).
[31] A. Thränhardt, S. Kuckenburg, A. Knorr, T. Meier, and S. W. Koch, Phys. Rev. B **62**, 2706 (2000).
[32] H. Haug and S. W. Koch, *Quantum Theory of the Optical and Electronic Properties of Semiconductors: Fifth Edition* (World Scientific Publishing Company, 2009).
[33] S. Brem, M. Selig, G. Berghaeuser, and E. Malic, Scientific Reports **8**, 8238 (2018).
[34] A. Kiss, L. Szolnoki, and F. Simon, Sci. Rep. **6**, 22706 (2016).
[35] M. Selig, G. Berghäuser, A. Raja, P. Nagler, C. Schüller, T. F. Heinz, T. Korn, A. Chernikov, E. Malic, and A. Knorr, Nat. Commun. **7**, 13279 (2016).
[36] S. Brem, J. Zipfel, M. Selig, A. Raja, L. Waldecker, J. D. Ziegler, T. Taniguchi, K. Watanabe, A. Chernikov, and E. Malic, Nanoscale **11**, 12381 (2019).
[37] R. Wallauer, R. Perea-Causin, L. Münster, S. Zajusch, S. Brem, J. Güdde, K. Tanimura, K.-Q. Lin, R. Huber, E. Malic, and U. Höfer, Nano Lett. **21**, 5867 (2021).
[38] R. Rosati, K. Wagner, S. Brem, R. Perea-Causín, E. Wietek, J. Zipfel, J. D. Ziegler, M. Selig, T. Taniguchi, K. Watanabe, A. Knorr, A. Chernikov, and E. Malic, ACS Photonics **7**, 2756 (2020).
[39] D. Schmitt, J. P. Bange, W. Bennecke, A. AlMutairi, G. Meneghini, K. Watanabe, T. Taniguchi, D. Steil, D. R. Luke, R. T. Weitz, S. Steil, G. S. M. Jansen, S. Brem, E. Malic, S. Hofmann, M. Reutzel, and S. Mathias, Nature **608**, 499 (2022).
[40] R. Rosati, R. Perea-Causín, S. Brem, and E. Malic, Nanoscale **12**, 356 (2020).
[41] J. C. König-Otto, M. Mittendorff, T. Winzer, F. Kadi, E. Malic, A. Knorr, C. Berger, W. A. de Heer, A. Pashkin, H. Schneider, M. Helm, and S. Winnerl, Phys. Rev. Lett. **117**, 087401 (2016).
[42] O. Hess and T. Kuhn, Phys. Rev. A **54**, 3347 (1996).
[43] M. Kulig, J. Zipfel, P. Nagler, S. Blanter, C. Schüller, T. Korn, N. Paradiso, M. M. Glazov, and A. Chernikov, Phys. Rev. Lett. **120**, 207401 (2018).
[44] R. Rosati, K. Wagner, S. Brem, R. Perea-Causín, J. D. Ziegler, J. Zipfel, T. Taniguchi, K. Watanabe, A. Chernikov, and E. Malic, Nanoscale **13**, 19966 (2021).
[45] M. M. Glazov, Phys. Rev. Lett. **124**, 166802 (2020).
[46] K. Wagner, J. Zipfel, R. Rosati, E. Wietek, J. D. Ziegler, S. Brem, R. Perea-Causín, T. Taniguchi, K. Watanabe, M. M.

Glazov, E. Malic, and A. Chernikov, Phys. Rev. Lett. **127**, 076801 (2021).
[47] J. M. Fitzgerald, R. Rosati, and E. Malic, Sci. Adv. **11**, eaea3495 (2025).
[48] C. G. Bailey, N. P. Sloane, T. L. Leung, C. Liao, A. Mena, D. M. de Clercq, J. Yi, S. Palomba, M. P. Nielsen, D. R. McKenzie, T. W. Schmidt, D. R. McCamey, and A. W. Y. Ho-Baillie, ACS Nano **19**, 41864 (2025).
